# Influence of increased nutrient availability on biogenic volatile organic compound (BVOC) emissions and leaf anatomy of subarctic dwarf shrubs under climate warming and increased cloudiness

**DOI:** 10.1093/aob/mcac004

**Published:** 2022-01-13

**Authors:** Flobert Ndah, Hanna Valolahti, Michelle Schollert, Anders Michelsen, Riikka Rinnan, Minna Kivimäenpää

**Affiliations:** Department of Environmental and Biological Sciences, University of Eastern Finland, P.O. Box 1627, 70211, Kuopio, Finland; Terrestrial Ecology Section, Department of Biology, University of Copenhagen, Copenhagen Ø 2100, Denmark; Center for Permafrost (CENPERM), Department of Geosciences and Natural Resource Management, University of Copenhagen, Copenhagen K 1350, Denmark; Ramboll, Niemenkatu 73, 15140, Lahti, Finland; Terrestrial Ecology Section, Department of Biology, University of Copenhagen, Copenhagen Ø 2100, Denmark; Center for Permafrost (CENPERM), Department of Geosciences and Natural Resource Management, University of Copenhagen, Copenhagen K 1350, Denmark; Department of Ecological Science, Faculty of Science, Vrije Universiteit Amsterdam, 1081 HV Amsterdam, The Netherlands; Terrestrial Ecology Section, Department of Biology, University of Copenhagen, Copenhagen Ø 2100, Denmark; Center for Permafrost (CENPERM), Department of Geosciences and Natural Resource Management, University of Copenhagen, Copenhagen K 1350, Denmark; Terrestrial Ecology Section, Department of Biology, University of Copenhagen, Copenhagen Ø 2100, Denmark; Center for Permafrost (CENPERM), Department of Geosciences and Natural Resource Management, University of Copenhagen, Copenhagen K 1350, Denmark; Department of Environmental and Biological Sciences, University of Eastern Finland, P.O. Box 1627, 70211, Kuopio, Finland; Natural Resources Institute Finland, Juntintie 154, 77600 Suonenjoki, Finland

**Keywords:** Arctic, soil nutrients, temperature, climate change, tundra, terpenoid, *Empetrum hermaphroditum*, *Cassiope tetragona*, *Betula nana*

## Abstract

**Background and Aims:**

Climate change is subjecting subarctic ecosystems to elevated temperature, increased nutrient availability and reduced light availability (due to increasing cloud cover). This may affect subarctic vegetation by altering the emissions of biogenic volatile organic compounds (BVOCs) and leaf anatomy. We investigated the effects of increased nutrient availability on BVOC emissions and leaf anatomy of three subarctic dwarf shrub species, *Empetrum hermaphroditum*, *Cassiope tetragona* and *Betula nana*, and if increased nutrient availability modifies the responses to warming and shading.

**Methods:**

Measurements of BVOCs were performed *in situ* in long-term field experiments in the Subarctic using a dynamic enclosure system and collection of BVOCs into adsorbent cartridges analysed by gas chromatography–mass spectrometry. Leaf anatomy was studied using light microscopy and scanning electron microscopy.

**Key Results:**

Increased nutrient availability increased monoterpene emission rates and altered the emission profile of *B. nana*, and increased sesquiterpene and oxygenated monoterpene emissions of *C. tetragona*. Increased nutrient availability increased leaf tissue thicknesses of *B. nana* and *C. tetragona*, while it caused thinner epidermis and the highest fraction of functional (intact) glandular trichomes for *E. hermaphroditum*. Increased nutrient availability and warming synergistically increased mesophyll intercellular space of *B. nana* and glandular trichome density of *C. tetragona*, while treatments combining increased nutrient availability and shading had an opposite effect in *C. tetragona*.

**Conclusions:**

Increased nutrient availability may enhance the protection capacity against biotic and abiotic stresses (especially heat and drought) in subarctic shrubs under future warming conditions as opposed to increased cloudiness, which could lead to decreased resistance. The study emphasizes the importance of changes in nutrient availability in the Subarctic, which can interact with climate warming and increased cloudiness effects.

## INTRODUCTION

The Arctic and Subarctic regions have experienced rapid climate change, with a temperature increase of ~1 °C over the last three decades, and the projected increase in mean annual temperature will be ~4 °C by mid-century (IPCC, 2018; Overland *et al.*, 2019). Rising air temperatures cause range shifts in vegetation (mainly increases in shrub abundance and height; Vowles and Björk, 2019; Myers-Smith *et al.*, 2020), permafrost thawing and increased release of greenhouse gases and soil mineralization rates, and are coupled to changes in cloud cover (Pearson *et al.*, 2013; IPCC, 2018). Increased soil organic matter mineralization rates caused by warming can lead to increased nutrient availability in the soil because of the release of nutrients bound to accumulated soil organic matter (Rustad *et al.*, 2001). In addition, the projected arctic summer air temperature increase is expected to accelerate the melting of the arctic ice cover, potentially leading to increased cloud cover due to atmospheric accumulation of aerosol particles from the ice-free surface as glaciers retreat (Kecorius *et al.*, 2019; Overland *et al.*, 2019).

The projected future changes in environmental conditions may affect subarctic vegetation, and thereby alter the emissions of biogenic volatile organic compounds (BVOCs) (Laothawornkitkul *et al.*, 2009). Plants emit substantial amounts of their photosynthetically assimilated carbon to the atmosphere as BVOCs (Peñuelas and Llusià, 2003) and the Subarctic region is considered a significant source of BVOCs (Tiiva *et al.*, 2008; Faubert *et al.*, 2010; Schollert, 2015; Tang *et al.*, 2016). BVOCs serve as a defence strategy against abiotic and biotic stresses (Holopainen and Gershenzon, 2010; Loreto and Schnitzler, 2010) and play an important role in plant-to-plant and plant-to-insect communication (Dicke and Baldwin, 2010; Heil and Karban, 2010). BVOCs can also be involved in complex atmospheric processes, which can feed back both negatively and positively on the climate system (Kulmala *et al.*, 2013). BVOCs play a key role in secondary organic aerosol formation, with indirect and direct effects on the emissions and atmospheric lifetime of greenhouse gases (Peñuelas and Llusià, 2003; Ehn *et al.*, 2014). Secondary organic aerosols enhance the formation of cloud condensation nuclei, resulting in increased cloudiness and radiative reflection with a net cooling effect (Shindell *et al.*, 2009; IPCC, 2013; Scott *et al.*, 2014). BVOC emissions are modified by changes in the environment, including global-change-related phenomena (Ormeño and Fernandez, 2012). Changes in nutrient availability, rising temperature and increased cloudiness are therefore expected to affect BVOC emissions.

The synthesis of carbon-based metabolites, such as terpenoids, a large group of BVOCs, is influenced by carbon and nutrient balance in the plant’s environment based on the carbon–nutrient balance hypothesis and the growth–differentiation balance hypothesis (Bryant *et al.*, 1983; Lorio, 1986; Herms and Mattson, 1992). Plants growing in soils with low nutrient availability tend to allocate more of an abundant resource, such as carbon, to the synthesis of defensive compounds, favouring the production and emission of terpenoids at the expense of growth (Bryant *et al*, 1983; Ormeño and Fernandez, 2012). The opposite holds for nutrient-rich soils, where excess carbon is allocated to growth rather than to the synthesis of terpenoids (Bryant *et al*, 1983; Lorio, 1986; Herms and Mattson, 1992; Ormeño and Fernandez, 2012). Unlike climate-related factors such as increased temperature, the effects of increased soil nutrient availability on BVOC emissions are not well documented and can be contrasting (Peñuelas and Staudt, 2010 and references therein; Ormeño and Fernandez, 2012). It is largely unknown how BVOC emissions from arctic and subarctic plants respond to increased soil nutrient availability.

Climate warming is likely to increase BVOC emissions due to their high temperature responsiveness in the Arctic and Subarctic (Tiiva *et al.*, 2008; Faubert *et al.*, 2010; Valolahti *et al.*, 2015; Lindwall *et al.*, 2016; Tang *et al.*, 2016). These studies have shown that experimental warming by 2–4 °C in air, using open-top chambers, more than doubles total volatile organic compound emissions, as well as those of specific compound groups such as monoterpenes and sesquiterpenes. Increased cloudiness (often studied as shading) also has the potential to affect BVOC emissions due to the light dependency of the synthesis and emissions of many BVOCs (Laothawornkitkul *et al.*, 2009; Monson and Baldocchi, 2014). For example, 6 years of experimental shading decreased volatile organic compound emissions from arctic vegetation by 65 % (Kramshøj *et al.*, 2016). Furthermore, nutrient availability and climate are key determinants of terpenoid emission types and rates (Fernández-Martínez *et al*., 2018). A previous study showed that climate warming combined with leaf litter addition, alleviating nutrient limitation, substantially increased ecosystem-level BVOC emissions due to increased plant biomass in the Subarctic tundra where dwarf shrubs were the dominant plant group (Valolahti *et al.*, 2015). These observations, which originate from studies conducted at plant community scale, highlight the need to further elucidate the effects of combined factors on BVOC emissions of different species of subarctic dwarf shrubs.

Plants can alter their leaf anatomy as a means of acclimating to changes in environmental conditions and these alterations can in turn affect BVOC emissions. Several studies have reported leaf anatomical alterations in tropical, temperate and boreal species developed under different nutrient availabilities (e.g. Longstreth and Nobel, 1980; Jurik *et al.*, 1982; Bilkova *et al.*, 2016; Kivimäenpää *et al.*, 2016; Tian *et al.*, 2016). Experimental warming and changes in light intensity have also been shown to cause leaf anatomical alterations in tree species (Larcher, 2003; Luomala *et al.*, 2005; Hartikainen *et al*., 2009, 2020; Kivimäenpää *et al.*, 2017; Thitz *et al.*, 2017), as well as in subarctic dwarf shrubs (Schollert *et al*., 2015, 2017). For example, thick epidermis and spongy parenchyma, and a decrease in palisade/spongy parenchyma ratio in connection with warming while thin epidermis, and reduction in glandular trichome (storage structure of terpenoids and other secondary compounds; Loreto and Schnitzler 2010) density in connection with shading have been reported for (sub)arctic plant species (Schollert *et al.*, 2015). Thicker leaf and palisade and thinner adaxial epidermis explained increased isoprene emissions in the broadleaved deciduous shrubs *Salix arctica* and *Betula nana* (Schollert *et al.*, 2015). Kivimäenpää *et al.* (2016) reported an increase in terpenoid emissions due to increased density of terpene-storing structures in Scots pine (*Pinus sylvestris*) in response to high nutrient availability and warming.

Previous studies have reported the effects of increased soil nutrient availability, warming and shading (simulating increased cloudiness) on BVOC emissions of (sub)arctic plants (Rinnan *et al.*, 2011; Schollert *et al.*, 2015), but not the interactions. While the effects of warming and shading on leaf anatomy of (sub)arctic plants have also been reported (Schollert *et al.*, 2015), there is a lack of information on the effects of increased nutrient availability and the interactions with warming and shading. Thus, our study provides new information that allows the effects of these different factors to be teased apart. The aim of this study was to assess the effects of increased nutrient availability on shoot BVOC emissions and leaf anatomy of subarctic dwarf shrubs and how increased nutrient availability modifies the effects of warming and shading. We hypothesized that increased nutrient availability leads to reduced BVOC emissions and trichome densities due to increased carbon allocation to growth rather than carbon-based secondary compounds and defence structures (Bryant *et al.*, 1983; Lorio, 1986; Herms and Mattson, 1992). We also hypothesized that increased nutrient availability and warming synergistically increase shoot-level BVOC emissions as previously observed at ecosystem level (Valolahti *et al.*, 2015), and due to increased density of glandular trichomes to conserve moisture under warming (Guerfel *et al.*, 2009). Furthermore, we hypothesized that increased nutrient availability and shading synergistically decrease emissions of the light-dependent BVOCs and lead to thicker leaf tissues and reduced density of glandular trichomes (Jurik *et al.*, 1982; Bryant *et al.*, 1983; Laothawornkitkul *et al.*, 2009; Monson and Baldocchi, 2014).

## MATERIALS AND METHODS

### Study site and experimental design

The experimental site was located on subarctic tundra heath in Abisko, Northern Sweden (68°21′N, 18°49′E; 450 m a.s.l.) and has been maintained since 1989, being one of the longest ongoing climate change field experiments in the world (Havström *et al.*, 1993). The growing season in Abisko normally lasts from June to September; mean annual temperature and precipitation are 0.49 °C and 332 mm (2002–11), respectively (Callaghan *et al.*, 2013).

The experimental design used in this study had six treatments: control, warming, shading, fertilization (increased nutrient availability), fertilization + warming and fertilization + shading (Rinnan *et al.*, 2007). Each treatment was replicated in six blocks, yielding 36 plots of 120 × 120 cm in total. Fertilization was provided by addition of nitrogen, phosphorus and potassium (solution of NO_4_NO_3_, KH_2_PO_4_ and KCl was added at a rate of 10.0, 2.6 and 9.0 g m^−2^ year^−1^ of N, P and K, respectively) every June since 1990 (except 1993 and 1998) and half of this amount in 1989, corresponding to the expected increase in soil nutrient availability due to atmospheric inputs and enhanced nutrient availability under a warmer climate (Rinnan *et al.*, 2007). Warming was achieved using open-top polyethylene film tents, which increases the air temperature by 3–4 °C (Havström *et al.*, 1993). Shading, mimicking the expected future increase in cloud coverage (IPCC, 2013), was provided by using hessian cloth tents, which reduced the incoming photosynthetic photon flux density (PPFD) by 50–60 % (Ellebjerg *et al.*, 2008). The growing season in 2012 has been described in detail by Schollert *et al.* (2015). Both warming and shading tents were put up for the snow-free period every year since 1989. In 2012, the tents were up between 19 May and 5 September. The total experimental duration prior to our measurements was 23 years.

Individual shoots of three woody species dominant at the experimental site were chosen for BVOC and leaf anatomy sampling (one plant per species per plot), involving two evergreen low shrubs, *Empetrum nigrum ssp. hermaphroditum* Hagerup and *Cassiope tetragona*, and the deciduous low shrub *Betula nana.* Data on emission of BVOCs from control, shading and warming treatments were presented by Schollert *et al.* (2015), but are grouped differently in the current study. Data from fertilization, fertilization + warming and fertilization + shading treatments are presented here for the first time.

### BVOC collection

Measurements of BVOCs for individual shoots were done between 4 and 7 August 2012 (mean daytime temperature 12.9 ± 0.3 °C, PPFD 415.3 ± 7.74 µmol m^2^ s^−1^; Schollert *et al.*, 2015), using pre-cleaned (60 min, 120 °C) polyethylene terephthalate bags, with a volume of 1 L, as enclosures around the shoots. The enclosures increased air temperature on average by 1.3 °C. Air inlets and outlets were led through holes in the bag corners, and the holes were tightly wrapped around the air tubing to prevent BVOCs from ambient air entering the sampling bag. Incoming air, led through Teflon tubing, was purified using a charcoal filter for removal of particles and volatile organic compounds and an MnO_2_ scrubber to remove ozone. Filtered air was allowed to fill the bags and replace the air for 3 min before collection. Air was circulated in the bag using battery-operated pumps (12 V; Rietschle Thomas, Puchheim, Germany) with flow rates of 500 mL min^−1^ for incoming air and 200 mL min^−1^ for outgoing sample air. Collection time for each sample was 30 min, yielding a 6-L sample volume. Samples were collected in stainless steel cartridges (Markes International Ltd, Llantrisant, UK), containing 150 mg of Tenax TA and 200 mg Carbograph 1 TD. Cartridges were sealed after sampling using Teflon-coated brass caps and stored at 4 °C until analysis. Temperature and air humidity during sampling were recorded every minute using DS 1923 iButton Hygrochron temperature/humidity loggers (Maxim Integrated, San Jose, CA, USA) and photosynthetically active radiation (PAR) was recorded every 10 s using S-LIA-M003 Photosynthetic Light Smart Sensors connected to a HOBO Micro Station data logger (model H21-002, Onset Computer Corporation, Boston, MA, USA). In addition, blank samples were collected from empty polyethylene terephthalate bags in a similar manner. After collection, the plant shoots were harvested, and after air-drying the dry weight of green leaves and stems (including attached dead leaves, for *C. tetragona*) was measured.

We analysed BVOCs using a thermal desorber (UNITY2, Markes International Ltd, Llantrisant, UK) coupled with an ULTRA autosampler and gas chromatograph–mass spectrometer (GC–MS; 7890A series GC, 5975C inert MSD/DS Performance Turbo EI System, Agilent, Santa Clara, CA, USA). For further details of GC–MS analysis see Valolahti *et al.* (2015). We identified BVOCs using standard compounds (Fluka, Buchs, Switzerland) and mass spectra provided by the NIST library and quantified them using isoprene standard for isoprene, α-pinene standard for monoterpenes, 1,8-cineole for oxygenated monoterpenes, α-humulene for sesquiterpenes and (*E*)-2-hexenal for green leaf volatiles and benzenoids (volatile aromatic compounds).

Enhanced ChemStation software (MSD ChemStation, E.02.01.1177, Agilent Technologies, Inc., Santa Clara, CA, USA) was used for analysing the chromatograms, and compounds with identification quality >90 % compared with the National Institute of Standards and Technology (NIST) mass spectral library were included in the further analysis. Emission rates (*E*) (ng g^−1^ dry weight h^−1^) of each compound were calculated by the formula for dynamic enclosure measurements: *E* = [(*C*_out_ − *C*_in_) × *F*]/*m*_,_ where *C*_out_ and *C*_in_ are the concentrations of BVOCs in the outlet and inlet air, respectively, *F* is the flow rate into the enclosure and *m* is the leaf dry mass. We assumed *C*_in_ to be zero (Ortega and Helmig, 2008; Niinemets *et al.*, 2011). Emission rates calculated for the blank samples were subtracted from the plant emissions.

We standardized monoterpenes, oxygenated monoterpenes, sesquiterpenes, green leaf volatiles and benzenoids, which are temperature-dependent, to the temperature of 30 °C, and isoprene, which is both temperature- and light-dependent, to the temperature of 30 °C and a PAR level of 1000 µmol m^−2^ s^−1^ according to Guenther *et al.* (1993) to minimize effects due to variations in environmental conditions during sampling. A *β* coefficient of 0.09 °C^−1^ was used for monoterpenes (Guenther *et al.*, 1993; Schollert *et al.*, 2015), 0.18 °C^−1^ for sesquiterpenes (Rinnan *et al.*, 2011; Schollert *et al.*, 2015) and 0.1 °C^−1^ for green leaf volatiles and benzenoids as other volatile organic compounds (Guenther *et al.*, 2012).

### Leaf anatomy

Fully developed green leaves were collected on 12 August 2012 between 1300 and 1500 h for light microscopy and for scanning electron microscopy (SEM). We carefully detached three leaves with forceps from the main shoot for *B. nana*, five first leaves from different sides of the current year shoot for *E. hermaphroditum* and five leaves from different sides of the current year shoot, ~8 mm from the tip, for *C. tetragona*.

Two leaves per species per plot were used for light microscopy. After collection, leaves were immediately placed in cold 2.5 % (v/v) glutaraldehyde fixative in 0.1 m sodium cacodylate buffer and cut later the same day into smaller sections. For *B. nana*, 1.5 × 1.5-mm sections were taken close to the central midrib from one half of each of the two leaves, and for *C. tetragona* and *E. hermaphroditum* 1.5-mm long sections were cut from the middle part of the leaf. Sectioned samples were kept in cold fixative until further preparation. Post-fixation in osmium tetroxide, embedding in Epon, sectioning, staining and imaging for light microscopy were done as described in Schollert *et al.* (2015).

The following leaf anatomical parameters were determined for all three studied species: leaf thickness; epidermis thickness; palisade and spongy tissue thickness and their ratio; proportion of intercellular space in palisade and spongy tissues; and leaf index from shape for *E. hermaphroditum*, as described in Schollert *et al.* (2015). Leaf margins of *C. tetragona* and *E. hermaphroditum* are curved, forming a cavity in the middle of the leaves. Thus, we use the terminology outer versus inner surface for them, corresponding to upper versus lower surface in *B. nana*. Leaf thickness of *E. hermaphroditum* was measured at two different locations (Supplementary Data Fig. S1) and the average was taken as the final leaf thickness. The density of stomata and glandular trichomes (number per millimetre) was related to the length of the inner surface of *E. hermaphroditum*. The condition of the *E. hermaphroditum* glandular trichomes was categorized as follows: 0 = deteriorated; 1 = deteriorating, partly collapsed or vacuolated; 2 = intact (Supplementary Data Fig. S2). *Cassiope tetragona* samples had spiky trichomes and trichomes with a sac-like gland with several compartments (glandular trichomes) on a stalk. Most of the latter type of trichomes were collapsed or severely deteriorated. The density of two types of trichomes (number per millimetre) was quantified on the inner side of the leaf and the surface facing the stem (Schollert *et al.*, 2015). The number of stomata per millimetre was determined for the inner side of the leaf.

Two leaves per species were used for SEM analysis. For *B. nana*, the same leaves as those used for light microscopic analysis (another half) was used for SEM analysis. Surface samples were air-dried before cutting and mounting on aluminium stubs. Samples were coated with gold–platinum under vacuum (Automatic Sputter Coater B7341, Agar Scientific Ltd, Stansted, UK) then examined and photographed at 300 × 700 magnification using SEM (Philips XL30 ESEM-TMP, FEI Company, Eindhoven, the Netherlands). For *B. nana*, the density of stomata (number per square millimetre) was determined on the lower side and the density of glandular trichomes was determined on both sides of the leaves. For *C. tetragona*, the density of spiky trichomes (number per square millimetre) was determined as a leaf average from several pictures taken from different sides of the leaf. The outer surface of *E. hermaphroditum* had glandular trichomes at the narrow edges of the leaf, and their density was determined as number per millimetre.

### Statistical analysis

Statistical analyses were run using IBM SPSS Statistics version 27. Linear mixed-model analysis of variance (LMM ANOVA) was used to evaluate how fertilization affected BVOC emissions and leaf anatomy variables and modified the effects of warming and shading. The model included fertilization as one fixed factor (two levels: non-fertilized and fertilized) and the climate treatments (three levels: control, warming and shading) as the other fixed factor and block as a random factor. The Bonferroni test was used for the more detailed study of interactions with *P* value <0.1 (simple main effects, i.e. *post hoc* tests of interactions). The Bonferroni test was also used to examine differences between control, warming and shading in the absence of interaction, when the climate treatment factor was significant. The Shapiro–Wilk normality test was used to check that the data and model residuals were normally distributed. If the residuals were not normally distributed, logarithmic or square root transformations were made. In all analyses, *P* < 0.05 was considered as statistically significant and *P* < 0.1 as a tendency. Statistical analyses were run for both actual BVOC emission data (Supplementary Data Table S1) and standardized emissions.

Principal component analysis (PCA) in SIMCA 15.0.2 (Umetrics, Umeå, Sweden) was used to evaluate the effects of fertilization and its interaction with climate treatments on the BVOC emission profile of each plant species. PCA components were extracted using cross-validation, centring and unit-variance scaling of the variables (emission rates of individual compounds). The scores for the first two components were analysed using LMM ANOVA models similar to those described earlier. Correlations between leaf anatomy variables and BVOC emissions for each of the studied plant species were analysed using Spearman’s rank-order correlation test.

## RESULTS

### BVOC emissions

Emissions from all three studied plant species comprised sesquiterpenes, isoprene, benzenoids, green leaf volatiles, monoterpenes and oxygenated monoterpenes, except that no green leaf volatiles were detected in *C. tetragona* emissions. Emission rates of the individual compounds identified for each species are shown in Supplementary Data Tables S2–S7. The emission profile, consisting of the percentage contribution of each BVOC group to the total BVOC emissions from *E. hermaphroditum*, *C. tetragona* and *B. nana*, averaged across treatments, is shown in Supplementary Data Fig. S3. PCA evaluating the interactive effects of fertilization and climate treatments on the emission profile of the studied plant species showed no significant trends or differences (data not shown). Therefore, PCA is shown only for fertilization with significant effects on total BVOC groups/classes (see below).

**Fig. 1.**
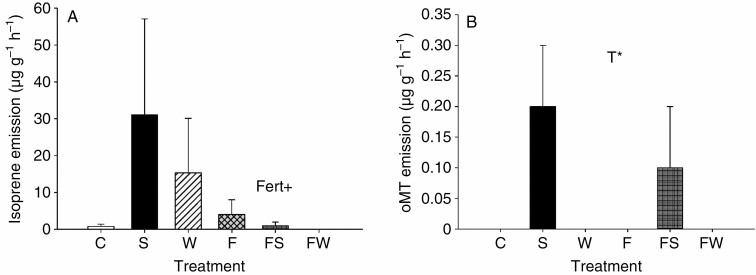
Standardized emissions (temperature of 30 °C, PAR of 1000 µmol m^−2^ s^−1^; Guenther *et al.*, 1993) (mean ± s.e., *n* = 5–6) of (A) isoprene and (B) oxygenated monoterpenes (oMT) from *E. hermaphroditum* under long-term control (C), shading (S), warming (W), fertilization (F), fertilization + shading (FS) and fertilization + warming (FW) treatments. Significant main effects of fertilization (Fert) and climate (T) treatments from mixed-model ANOVAs are indicated by * (*P* < 0.05) and + (*P* < 0.1). See main text for pairwise comparisons using the Bonferroni test.

For *E. hermaphroditum*, fertilization had no significant effect on emission rates or standardized emissions of any of the BVOC groups (Table 1, Supplementary Data Table S1), except that it tended to decrease standardized isoprene emission by 76 % (Fig. 1A). The emission profile, consisting of the individual compounds emitted from *E. hermaphroditum*, was not significantly affected by fertilization as PCA revealed no clear separation between fertilized and non-fertilized treatments (Supplementary Data Fig. S4). Shading increased emission rates and standardized emission of oxygenated monoterpenes by 2 and 12 %, respectively, compared with the other climate treatments (Fig. 1B, Supplementary Data Table S1; 0.05 < *P* < 0.1 for control versus shaded and warmed versus shaded treatment comparisons by the Bonferroni test). This increase was due to the increase in the emission and standardized emission of geranyl acetone, which was the only emitted oxygenated monoterpene and constituted 1 % of the total BVOC emissions (Supplementary Data Tables S2 and S3 and Fig. S3). There were no significant interactions between fertilization and the climate treatments for emissions or standardized emissions of any of the BVOC groups (Table 1). Sesquiterpenes, with copaene and α-cubebene as the dominant compounds, represented the largest proportion (38 %) of the total emissions (Supplementary Data Fig. S3). Isoprene constituted 37 % of the total emissions (Supplementary Data Fig. S3). Benzenoids constituted 13 % and green leaf volatiles 10 % of the total emissions, with benzaldehyde and *cis*-3-hexenyl acetate as the most emitted compounds, respectively, while monoterpenes constituted 1 % of the total emissions, with cymene being the most emitted monoterpene (Supplementary Data Fig. S3).

**Table 1.** Standardized emissions (µg g^−1^ h^−1^, mean ± s.e., *n* = 5–6) of isoprene, non-oxygenated monoterpenes, oxygenated monoterpenes, sesquiterpenes, green leaf volatiles and benzenoids from *E. hermaphroditum*, *C. tetragona* and *B. nana* under long-term control (C), shading (S), warming (W), fertilization (F), fertilization + shading (FS) and fertilization + warming (FW) treatments. T = climate treatment, Fert = fertilization, T × Fert = climate treatment × fertilization

Standardized emissions (µg g^−1^ h^−1^)							*P* value		
	C	S	W	F	FS	FW	T	Fert	T × Fert
*E. hermaphroditum*									
Non-oxygenated monoterpenes	0.1 ± 0.1	0.1 ± 0.1	0.0 ± 0.0	0.1 ± 0.1	0.1 ± 0.1	0.0 ± 0.0	0.574	0.834	0.979
Sesquiterpenes	5.5 ± 3.0	31.3 ± 19.2	1.6 ± 0.6	14.5 ± 13.7	5.3 ± 4.4	4.1 ± 3.8	0.406	0.359	0.483
Green leaf volatiles	0.4 ± 0.4	1.4 ± 1.4	0.8 ± 0.8	0.4 ± 0.4	0.8 ± 0.8	0.0 ± 0.0	0.753	0.476	0.842
Benzenoids	0.5 ± 0.5	1.2 ± 0.8	0.0 ± 0.0	1.2 ± 1.2	1.7 ± 1.7	0.0 ± 0.0	0.337	0.984	0.931
*C. tetragona*									
Non-oxygenated monoterpenes	0.1 ± 0.0	1.8 ± 1.0	0.6 ± 0.6	19.4 ± 19.3	2.5 ± 1.1	2.9 ± 2.0	0.603	0.101	0.756
Benzenoids	0.0 ± 0.0	2.1 ± 1.5	0.9 ± 0.9	5.0 ± 5.0	2.7 ± 1.5	2.2 ± 1.7	0.400	0.199	0.873
*B. nana*									
Isoprene	2.1 ± 1.7	7.5 ± 5.8	7.3 ± 5.0	0.4 ± 0.4	25.0 ± 25.0	0.3 ± 0.2	0.485	0.137	0.769
Oxygenated monoterpenes	0.2 ± 0.2	0.4 ± 0.4	0.4 ± 0.4	0.3 ± 0.2	0.2 ± 0.1	0.1 ± 0.1	0.927	0.686	0.478
Sesquiterpenes	2.6 ± 2.4	7.6 ± 6.8	11.6 ± 11.4	8.8 ± 8.1	9.8 ± 7.1	1.6 ± 1.4	0.703	0.863	0.711
Green leaf volatiles	45.8 ± 44.5	119.3 ± 117.0	56.4 ± 53.2	61.4 ± 58.8	16.3 ± 8.6	8.8 ± 6.4	0.913	0.823	0.832
Benzenoids	1.9 ± 1.9	0.7 ± 0.7	1.5 ± 1.5	2.5 ± 2.5	0.0 ± 0.0	0.0 ± 0.0	0.328	0.581	0.506

*P* values are from LMM ANOVA. The standardized emissions (minimizing the effects of variations caused by sampling environmental conditions) were calculated according to Guenther *et al.* (1993), i.e. actual emission rates were standardized to 30 °C and that of isoprene to a PAR of 1000 µmol m^−2^ s^−1^.

**Fig. 2.**
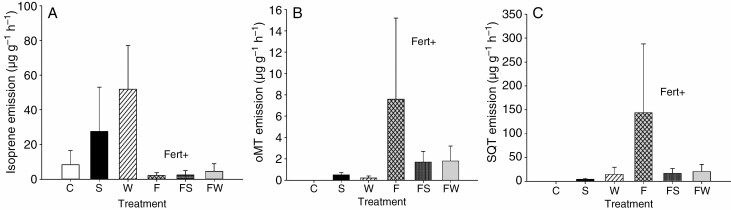
Standardized emissions (temperature of 30 °C, PAR of 1000 µmol m^−2^ s^−1^; Guenther *et al.*, 1993) (mean ± s.e., *n* = 5–6) of (A) isoprene, (B) oxygenated monoterpenes (oMT) and (C) sesquiterpenes (SQT) from *C. tetragona* under long-term control (C), shading (S), warming (W), fertilization (F), fertilization + shading (FS) and fertilization + warming (FW) treatments. Significant main effect of fertilization (Fert) from mixed models ANOVAs is indicated by + (*P* < 0.1).

For *C. tetragona*, fertilization tended to decrease isoprene emission rates and standardized emission by 73 and 64 %, respectively (Fig. 2A, Supplementary Data Table S1), and increase the standardized emission of oxygenated monoterpenes and sesquiterpenes by 275 and 140 %, respectively (Fig. 2B, C), with no significant main effects on emission rates or standardized emissions of other BVOC groups (Table 1, Supplementary Data Table S1). A PCA of the emission profile of *C. tetragona* revealed no clear separation between fertilized and non-fertilized treatments (Supplementary Data Fig. S5). There were no significant interactions between fertilization and the climate treatments on emissions or standardized emissions of any of the BVOC groups (Table 1, Supplementary Data Table S1). Sesquiterpenes, with α-eudesmol, γ-eudesmol and cadinene as the major compounds, constituted 35 % of the total emissions (Supplementary Data Fig. S3). Monoterpenes constituted 24 % of the total emissions, with cymene and limonene being the most abundant monoterpenes (Supplementary Data Fig. S3). Isoprene constituted 21 % and oxygenated monoterpenes 10 % of the total emissions; 1,8-cineole, terpinen-4-ol and α-terpineol were the most abundant oxygenated monoterpenes while benzenoids constituted 9 % of the total emissions, with benzaldehyde as the dominant compound (Supplementary Data Fig. S3).

**Fig. 3.**
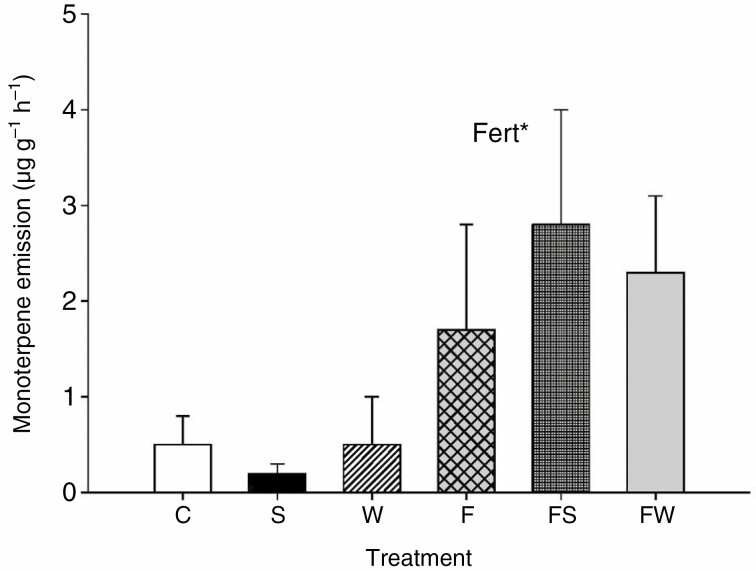
Standardized emission (temperature of 30 °C; Guenther *et al.*, 1993) (mean ± s.e., *n* = 5–6) of non-oxygenated monoterpenes from *B. nana* under long-term control (C), shading (S), warming (W), fertilization (F), fertilization + shading (FS) and fertilization + warming (FW) treatments. Significant main effect of fertilization (Fert) from mixed-model ANOVA is indicated by * (*P* < 0.05).

**Fig. 4.**
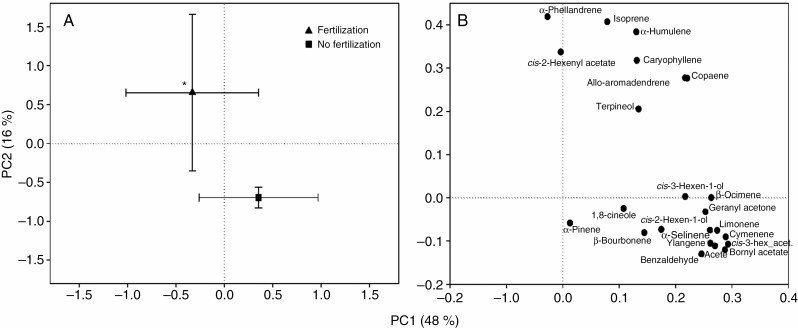
PCA of the emissions of individual BVOCs from *B. nana*. (A) Mean scores (±s.e; *n* = 15–16) for fertilization and no fertilization treatments on PC1 and PC2 and (B) the corresponding loading plot. PC1 and PC2 explained 48 and 16 % of the variation, respectively. Significant main effect of fertilization is shown (PC2, LMM ANOVA, *P < 0.05). Abbreviations: cis-3-hex_acet. = *cis*-3-hexenyl acetate; aceto. = acetophenone.

For *B. nana*, fertilization significantly increased monoterpene emission rates and standardized emissions by 372 and 269 %, respectively (Fig. 3, Supplementary Data Table S1), and had no significant main effects on other BVOC groups (Table 1, Supplementary Data Table S1). PCA revealed a significant effect of fertilization on the emission profile of *B. nana* along PC2 (Fig. 4). The emissions from the fertilized treatments were characterized by higher relative contributions of isoprene, *cis*-2-hexenyl acetate (green leaf volatile), α-phellandrene and terpineol (monoterpenes), and the sesquiterpenes copaene, caryophyllene, α-humulene and allo-aromadendrene compared with treatments without fertilization (Fig. 4). There were no significant interactions between fertilization and the climate treatments for emissions or standardized emissions of any of the BVOC groups (Table 1, Supplementary Data Table S1). Total green leaf volatiles, which constituted 86 % of the total emissions, with *cis*-3-hexen-1-ol and *cis*-3-hexenyl acetate as the dominant compounds (Supplementary Data Fig. S3), were not affected by the treatments. Isoprene constituted 5 % of the total emissions while monoterpenes and sesquiterpenes constituted 3 % each of the total emissions, with α-phellandrene and allo-aromadendrene as the dominant compounds, respectively (Supplementary Data Fig. S3). Benzenoids constituted 2 % and oxygenated monoterpenes 1 % of the total emissions, with benzaldehyde and geranyl acetone as the dominant compounds, respectively (Supplementary Data Fig. S3).

### Leaf anatomy

For *E. hermaphroditum*, glandular trichome condition was best in fertilization treatments (Table 2). Fertilization decreased outer epidermis thickness by 10 % and tended to decrease palisade parenchyma thickness by 13 % while increasing the intercellular space in the palisade tissue from 18 to 23 % and the total intercellular space from 41 to 46 % (Table 2). There was a significant main effect of climate treatment on the total intercellular space as both warming and shading increased the total intercellular space by 12 and 11 %, respectively, compared with the control treatment, but this difference was only marginally significant (Table 2; *P* = 0.08 for control versus warmed and *P* = 0.1 for control versus shaded treatment comparisons by the Bonferroni test). There were no significant interactions between fertilization and climate treatments for any of the leaf anatomy variables (Table 2).

**Table 2.** Leaf anatomy variables of *E. hermaphroditum* under long-term control (C), shading (S), warming (W), fertilization (F), fertilization + shading (FS) and fertilization + warming (FW) treatments. T = climate treatment; Fert = fertilization; T x Fert = climate treatment x fertilization

	Leaf anatomy						*P* value		
	C	S	W	F	FS	FW	T	Fert	T × Fert
*E. hermaphroditum*									
Shape index	2.0 ± 0.1	2.2 ± 0.2	2.3 ± 0.2	2.2 ± 0.1	2.3 ± 0.2	2.2 ± 0.1	0.586	0.833	0.729
Leaf thickness (µm)	236.3 ± 11.9	231.0 ± 7.3	246.6 ± 18.0	229.5 ± 13.2	209.9 ± 10.7	218.9 ± 15.6	0.581	0.103	0.732
Glandular trichome density on outer surface (no. per mm)	6.1 ± 0.4	5.9 ± 0.9	5.9 ± 0.3	5.5 ± 0.2	5.9 ± 0.3	6.4 ± 0.3	0.753	0.979	0.496
Glandular trichome density on inner surface (no. per mm)	3.1 ± 0.6	3.0 ± 0.5	2.1 ± 0.2	3.3 ± 0.4	2.9 ± 0.3	3.6 ± 0.5	0.808	0.145	0.182
Glandular trichome condition	0.8 ± 0.4	0.6 ± 0.2	0.5 ± 0.3	1.5 ± 0.2	1.7 ± 0.2	1.5 ± 0.2	0.762	**<0.001**	0.769
Stomata density on inner surface (no. per mm)	2.5 ± 0.3	2.3 ± 0.3	2.2 ± 0.3	3.0 ± 0.6	3.0 ± 0.4	2.6 ± 0.6	0.713	0.135	0.947
Outer epidermis thickness (µm)	17.0 ± 0.7	18.0 ± 1.1	18.1 ± 1.4	15.9 ± 1.4	17.1 ± 1.1	14.7 ± 0.6	0.509	**0.050**	0.467
Inner epidermis thickness (µm)	10.9 ± 0.5	10.5 ± 0.2	11.0 ± 0.4	11.0 ± 0.8	9.6 ± 0.4	10.4 ± 0.3	0.194	0.277	0.607
Palisade parenchyma thickness (µm)	85.4 ± 7.3	77.2 ± 8.2	83.6 ± 9.7	77.0 ± 4.9	69.0 ± 6.7	68.2 ± 5.1	0.547	**0.091**	0.858
Spongy parenchyma thickness (µm)	90.8 ± 4.9	89.6 ± 9.6	95.7 ± 8.4	90.6 ± 6.4	80.7 ± 5.9	90.9 ± 9.9	0.553	0.410	0.848
Palisade/spongy parenchyma ratio	1.0 ± 0.1	0.9 ± 0.1	0.9 ± 0.1	0.9 ± 0.1	0.9 ± 0.1	0.8 ± 0.1	0.651	0.382	0.910
Intercellular space, palisade (%)	14.6 ± 2.3	18.3 ± 2.4	19.6 ± 3.8	21.2 ± 2.0	27.3 ± 1.6	21.8 ± 2.8	0.184	**0.010**	0.439
Intercellular space, spongy (%)	63.2 ± 2.7	62.5 ± 2.0	67.4 ± 2.9	66.5 ± 3.8	67.8 ± 2.9	67.6 ± 0.9	0.583	0.210	0.653
Intercellular space total (%)	38.2 ± 1.9	42.0 ± 1.3	43.7 ± 2.2	42.8 ± 2.9	48.2 ± 2.3	47.1 ± 0.4	**0.044**	**0.010**	0.788

*P* values from LMM ANOVA are shown, with *P* < 0.1 emboldened. See main text for pairwise comparisons by Bonferroni test.

For *C. tetragona*, fertilization significantly increased inner epidermis thickness by 7 % and tended to reduce glandular trichome density by 0.4 trichomes per millimetre and stomata density by 0.6 stomata per millimetre of inner leaf surface length (Table 3). Spiky trichomes facing the stem were absent in the fertilized treatments but not in the non-fertilized treatments (Table 3). There was a significant fertilization × climate treatment interaction on the density of glandular trichomes facing the stem (Table 3). Fertilization reduced the density of glandular trichomes facing the stem by 1.6 trichomes per millimetre in control and 2.8 trichomes per millimetre in shading treatments (*P* = 0.033 for control versus fertilization and *P* = 0.003 for shading versus fertilization + shading comparisons by the Bonferroni test) but not in warming treatments (*P* > 0.1 for the warming versus fertilization + warming comparison by the Bonferroni test; Table 3). In the absence of fertilization, there were no significant effects of shading or warming on the density of glandular trichomes facing the stem (*P* > 0.1 for control versus warming and control versus shading comparisons by the Bonferroni test, Table 3). In the presence of fertilization, warming increased glandular trichome density 2-fold compared with control and shading treatments (*P* = 0.002 for fertilization versus fertilization + warming and *P* = 0.002 for fertilization + shading versus fertilization + warming comparisons by the Bonferroni test). Warming increased spiky trichome density by 0.4 trichomes per millimetre of inner leaf surface length compared with shading (*P* = 0.012 for shaded versus warmed treatments comparison by the Bonferroni test, Table 3) and tended to decrease stomata density by 0.8 mm^−1^ inner leaf surface length and intercellular space of spongy tissue 0.7 to 0.6 % compared with control treatments (Table 3).

**Table 3.** Leaf anatomy variables of *C. tetragona* under long-term control (C), shading (S), warming (W), fertilization (F), fertilization + shading (FS) and fertilization + warming (FW) treatments. T = climate treatment; Fert = fertilization; T x Fert = climate treatment x fertilization

Leaf anatomy							*P* value		
	C	S	W	F	FS	FW	T	Fert	T × Fert
*C. tetragona*									
Leaf thickness (µm)	196.7 ± 9.3	192.6 ± 13.2	182.8 ± 12.9	207.5 ± 10.6	189.8 ± 10.1	184.2 ± 16.3	0.319	0.702	0.836
Glandular trichome density on inner surface (no. per mm)	1.6 ± 0.3	1.3 ± 0.3	1.5 ± 0.3	0.9 ± 0.3	1.2 ± 0.2	1.0 ± 0.3	0.989	**0.069**	0.401
Glandular trichome density on surface against stem (no. per mm)	4.7 ± 0.4	5.5 ± 0.5	6.1 ± 0.9	3.3 ± 0.6	2.8 ± 0.8	6.1 ± 0.7	**0.001**	**0.005**	**0.035**
Spiky trichome density on surface (number per mm^2^)	300.8 ± 28.7	202.6 ± 47.2	210.7 ± 44.6	201.9 ± 40.6	170.3 ± 47.1	221.7 ± 58.0	0.142	0.156	0.280
Spiky trichome density on inner surface (no. per mm)	5.0 ± 2.1	3.2 ± 0.8	11.1 ± 3.3	4.4 ± 1.6	4.5 ± 2.5	9.2 ± 3.6	**0.012**	0.324	0.855
Spiky trichome density on surface against stem (no. per mm)	0.4 ± 0.2	0.2 ± 0.1	0.9 ± 0.8	0.0 ± 0.0	0.0 ± 0.0	0.0 ± 0.0	0.641	**0.071**	0.747
Stomata density on inner surface (no. per mm)	3.5 ± 0.3	2.9 ± 0.4	2.8 ± 0.5	3.0 ± 0.5	2.2 ± 0.3	2.1 ± 0.2	**0.087**	**0.065**	0.918
Outer epidermis thickness (µm)	21.0 ± 1.1	21.5 ± 1.1	21.6 ± 1.1	21.8 ± 1.3	20.5 ± 0.7	18.6 ± 1.0	0.492	0.223	0.217
Inner epidermis thickness (µm)	10.8 ± 0.5	10.7 ± 0.3	10.7 ± 0.3	11.4 ± 0.3	12.1 ± 0.5	11.0 ± 0.4	0.368	**0.024**	0.411
Palisade parenchyma thickness (µm)	83.0 ± 5.5	82.9 ± 8.4	83.9 ± 10.6	87.8 ± 5.5	75.0 ± 6.6	78.5 ± 13.7	0.739	0.688	0.731
Spongy parenchyma thickness (µm)	85.4 ± 4.9	80.6 ± 4.7	70.5 ± 6.5	88.1 ± 7.6	85.4 ± 5.5	79.3 ± 3.6	0.127	0.206	0.807
Palisade/spongy parenchyma ratio	1.0 ± 0.1	1.0 ± 0.1	1.3 ± 0.3	1.1 ± 0.1	0.9 ± 0.1	1.0 ± 0.2	0.477	0.254	0.548
Intercellular space, palisade (%)	0.2 ± 0.0	0.2 ± 0.0	0.1 ± 0.0	0.2 ± 0.0	0.2 ± 0.0	0.2 ± 0.1	0.986	0.523	0.105
Intercellular space, spongy (%)	0.7 ± 0.0	0.7 ± 0.0	0.6 ± 0.0	0.7 ± 0.0	0.7 ± 0.0	0.6 ± 0.0	**0.070**	0.705	0.368
Intercellular space total (%)	0.4 ± 0.0	0.4 ± 0.0	0.4 ± 0.0	0.4 ± 0.0	0.4 ± 0.0	0.4 ± 0.0	0.548	0.399	0.851

*P* values from LMM ANOVA are shown, with *P* < 0.1 emboldened. See main text for pairwise comparisons by Bonferroni test.

For *B. nana*, fertilization significantly increased leaf thickness by 10 %, lower and upper epidermis thickness by 4 and 14 %, respectively, and spongy parenchyma thickness by 12 % (Table 4). There was a marginally significant fertilization × climate treatment interaction on the intercellular space of spongy tissue and total intercellular space (Table 4). Fertilization increased the intercellular space of spongy tissue under warming from 63 to 75 % (*P* = 0.026 for the warming versus fertilization + warming warming comparison by the Bonferroni test) but not in control and shading treatments (*P* > 0.1 for control versus fertilization and shading versus fertilization + shading comparisons by the Bonferroni test; Table 4). In the absence of fertilization, shading increased the intercellular space of spongy tissue compared with control from 60 to 75 % and tended to increase it compared with warming treatments from 63 to 75 % (*P* = 0.019 for control versus shading and *P* = 0.080 for warming versus shading comparisons by the Bonferroni test; Table 4). In the presence of fertilization, no significant effects of shading or warming were found on the intercellular space of spongy tissue. Changes in the intercellular space of spongy tissue accounted for the observed changes in total intercellular space. Warming increased stomatal density by 8 % while shading decreased it by 19 % (*P* = 0.021 for control versus warmed and control versus shaded treatment comparisons by the Bonferroni test). Shading decreased the palisade/spongy parenchyma ratio (0.5) compared with control (0.7) (*P* = 0.019 for control versus shaded treatments comparison by the Bonferroni test) and tended to decrease palisade parenchyma thickness by 21 % compared with warming (*P* = 0.097 for shaded versus warmed treatments comparison by the Bonferroni test).

**Table 4.** Leaf anatomy variables of *B. nana* under long-term control (C), shading (S), warming (W), fertilization (F), fertilization + shading (FS) and fertilization + warming (FW) treatments. T = climate treatment; Fert = fertilization; T x Fert = climate treatment x fertilization

Leaf anatomy							*P* value		
	C	S	W	F	FS	FW	T	Fert	T × Fert
*B. nana*									
Leaf thickness (µm)	210.8 ± 11.3	202.0 ± 14.1	215.2 ± 9.2	226.2 ± 8.4	222.3 ± 9.9	246.4 ± 9.7	0.210	**0.015**	0.742
Glandular trichome density (no. per mm^2^)	6.6 ± 0.8	4.4 ± 0.7	3.9 ± 0.9	3.9 ± 0.5	4.2 ± 0.8	4.5 ± 1.2	0.392	0.301	0.153
Stomata density (no. per mm^2^)	116.0 ± 5.7	96.7 ± 10.9	123.8 ± 12.4	115.4 ± 13.9	91.7 ± 10.7	124.9 ± 7.4	**0.020**	0.833	0.964
Upper epidermis thickness (µm)	26.4 ± 0.9	27.6 ± 1.3	22.9 ± 2.2	28.7 ± 1.5	29.4 ± 1.1	29.6 ± 0.8	0.260	**0.003**	0.160
Lower epidermis thickness (µm)	14.1 ± 0.7	15.8 ± 0.6	13.2 ± 1.1	15.4 ± 1.1	16.8 ± 1.1	16.3 ± 1.2	0.164	**0.035**	0.433
Palisade parenchyma thickness (µm)	67.5 ± 6.3	55.4 ± 11.6	67.4 ± 1.3	76.1 ± 8.4	61.7 ± 5.9	80.8 ± 6.6	**0.072**	0.109	0.872
Spongy parenchyma thickness (µm)	97.0 ± 4.9	103.6 ± 6.0	104.4 ± 7.7	104.4 ± 2.6	114.1 ± 7.9	123.4 ± 5.9	0.126	**0.024**	0.636
Palisade/spongy parenchyma ratio	0.7 ± 0.1	0.5 ± 0.1	0.7 ± 0.1	0.7 ± 0.1	0.5 ± 0.0	0.7 ± 0.1	**0.020**	0.783	0.926
Intercellular space palisade (%)	17.5 ± 2.1	28.8 ± 3.2	21.8 ± 2.5	21.3 ± 3.4	21.8 ± 1.3	19.2 ± 2.9	**0.089**	0.382	0.157
Intercellular space, spongy (%)	60.4 ± 2.7	75.0 ± 2.7	63.4 ± 4.4	68.5 ± 4.0	70.5 ± 2.4	74.5 ± 3.7	**0.086**	**0.098**	**0.083**
Intercellular space, total (%)	40.1 ± 2.4	57.2 ± 2.6	45.2 ± 3.8	50.0 ± 3.3	52.7 ± 2.8	49.8 ± 2.9	**0.011**	0.196	**0.089**

*P* values from LMM ANOVA are shown, with *P* < 0.1 emboldened. See main text for pairwise comparisons by Bonferroni test.

### Correlation between leaf anatomy and BVOC emissions

Spearman’s rank-order correlation test was used to evaluate the relationships between leaf anatomy variables and emission rates of the BVOC groups. For all the studied species, no significant correlations were found between leaf anatomy variables and BVOC emissions (data not shown).

## DISCUSSION

### *Higher nutrient availability increased BVOC emissions of* B. nana *and* C. tetragona *and altered leaf anatomy of all studied species*

Contrary to our expectations, increased nutrient availability did not decrease BVOC emissions but increased monoterpene emissions of *B. nana* and tended to increase oxygenated monoterpene and sesquiterpene emissions of *C. tetragona*. In a sampling conducted at the same site 5 years earlier, 18 years from the beginning of the experiment, Rinnan *et al.* (2011) found that fertilization caused no statistically significant change in BVOC emissions from the same shrubs. Valolahti *et al.* (2015) reported that ecosystem-level terpenoid emissions from dwarf-shrub-dominated subarctic tundra were not affected by litter addition increasing nutrient availability after 11 and 13 years of experimental manipulation, but litter addition enhanced the BVOC emissions from warming treatment. The responses observed in this study suggest that more than two decades of increased nutrient availability starts to affect BVOC emissions of individual dwarf shrub species, which highlights the importance of long-term experiments.

We observed compound-specific responses in the emission profile of *B. nana* to increased nutrient availability. The relative contributions of isoprene, *cis*-2-hexenyl acetate (green leaf volatile), α-phellandrene and terpineol (monoterpenes), and the sesquiterpenes allo-aromadendrene, copaene, caryophyllene and α-humulene were higher in the fertilized treatments than in the non-fertilized treatments. In this study, increased emissions of individual compounds or compound groups in response to increased nutrient availability may be due to the influence of increased nutrient availability on biochemical processes controlling their synthesis and emissions. Nitrogen, for example, could promote electron transport rate and CO_2_ fixation, which provide energy and substrates favouring isoprene, monoterpene and sesquiterpene synthesis and emissions (Ormeño and Fernandez, 2012). This is typical in non-storing species, which frequently feature stronger and faster short-term responses to environmental factors due to the absence of terpenoid reservoirs that buffer the direct influence of the environment (Blanch *et al.*, 2007; Ormeño *et al.*, 2011), but can also occur in storing species since all carbon-based secondary metabolites ultimately depend on CO_2_ fixation (Ormeño and Fernandez, 2012). A large percentage of monoterpene emissions from subarctic ecosystems has been shown to be derived from *de novo* biosynthesis, further highlighting the crucial role of factors affecting biochemical processes involved in terpenoid biosynthesis and emissions (Ghirardo *et al.*, 2020).

Similar to BVOC emissions, increased nutrient availability caused contrasting responses in anatomy between the studied species, possibly highlighting their different acclimation strategies. We observed a decrease in trichome density of *C. tetragona* in response to increased nutrient availability. This may be indicative of more carbon investment in growth at the expense of the formation of defence structures such as trichomes (Bryant *et al.*, 1983; Hems and Mattson, 1992) but may also be an indirect consequence of higher nutrient availability increasing leaf area or size. Alternatively, the glandular trichome density may have been constrained by stronger nitrogen limitation at later stages of leaf growth (Bilkova *et al.* 2016). Tian *et al.* (2016) showed that leaf thickness can be controlled by soil nutrients and increases significantly with an increase in palisade and spongy mesophyll thickness. Sims *et al.* (1998) also showed that higher nitrogen supply increased leaf thickness due to an increase in mesophyll cell layers, with minimal effects on epidermal cell layers. Therefore, the observed increase in the overall leaf thickness of *B. nana* may be due to increases in spongy mesophyll thickness and epidermis thickness in response to increased nutrient availability. For *C. tetragona*, inner epidermis thickness increased in response to increased nutrient availability but not sufficiently to cause a significant increase in overall leaf thickness. Decreased stomatal density of *C. tetragona* may be an indirect consequence of higher nutrient availability increasing epidermis cell size, and not due to reduction in stomatal initiation. For *E. hermaphroditum*, the outer epidermis was thinner under fertilization than without it. The outer epidermis growth in *E. hermaphroditum* may have been constrained by shortages of some other resources despite increased nutrient availability. Samples in this study were collected in the late growing season (August) and glandular trichomes in the studied plant species had deteriorated as a normal process of leaf ageing (Valkama *et al.*, 2004). The high proportion of intact glandular trichomes under increased nutrient availability in *E. hermaphroditum* may be due to a delay in leaf senescence or ageing under increased fertilization (Zhang *et al.*, 2010), thereby improving the chemical defence capacity of *E. hermaphroditum* against biotic stresses at the end of the growing season (Muravnik and Shavarda, 2012).

The leaf tissue or epidermis thickness has been suggested to control BVOC emissions directly or indirectly by modifying diffusion pathways, residence time of BVOCs within the leaf and within-leaf metabolism (Hartikainen *et al.*, 2009; Niinemets *et al.*, 2014; Schollert *et al.*, 2015), but we found no statistically significant links between these variables. In this study, we found no connection between trichomes and BVOC emission rates, though trichome condition and number are associated with BVOC emission rates (Mofikoya *et al.*, 2018). This may be due to the late season and disruption of storage structures or to the glandular trichomes of these species acting primarily as storage for other secondary chemicals. Changes in light and photosynthesis-dependent isoprene emissions of *C. tetragona* and *E*. *hermaphroditum* actually suggest that fertilization had greater effects on photosynthesis-dependent *de novo* emissions and emissions from temporary or non-storage pools than on emissions from long-term storage pools such as those contained in glandular trichomes (Laothawornkitkul *et al.*, 2009; Lindwall *et al.*, 2015; Ghirardo *et al.*, 2020).

### *Increased nutrient availability modifies* C. tetragona *and* B. nana *anatomy responses caused by warming*

Contrary to results from ecosystem-level experiments, which reported strong effects of higher nutrient availability (resulting from litter addition) under warming (Faubert *et al.*, 2010; Valolahti *et al.*, 2015), plant shoot BVOC emissions in this study were barely affected by treatments combining increased nutrient availability with warming. Together, the studies may imply that when increased nutrient availability enhances the effects of warming on tundra ecosystem BVOC emissions, the emissions from soil microbes or litter decomposition are affected more than emission rates of individual plants. Nevertheless, increased nutrient availability modified anatomical responses to warming. In the presence of increased nutrient availability, warming caused a 2-fold increase in the density of glandular trichomes facing the stem of *C. tetragona*. Increased trichome densities and a decrease in the stomatal density of *C. tetragona* in response to warming irrespective of increased nutrient availability suggest a strategy to enhance their protection capacity against water deficit or heat (Valkama *et al.*, 2004; Luomala *et al.*, 2005; Guerfel *et al.*, 2009; Schollert *et al.*, 2017). The tendency of increased nutrient availability to increase the intercellular space under warming for *B. nana* may indicate a structural acclimation to cool the leaves by increasing transpiration (Kivimäenpää *et al.*, 2017). Increased stomata density of *B. nana* can be linked to an increase in cell differentiation under relatively warm conditions and can also indicate increased transpiration (Luomala *et al.*, 2005; Tian *et al.*, 2016). Potential acclimation to increased transpiration under warming was also observed in *E*. *hermaphroditum* as higher intercellular space but was not modified by nutrient availability. The results suggest that increased nutrient availability may increase the capacity of *C. tetragona* and *B. nana* to prevent water deficit or heat stress in warmer climatic conditions. The observed changes in stomatal density (a decrease for *C. tetragona* and an increase for *B. nana*) under warming may be unlikely to have affected BVOC emissions due to lack of any significant correlations between these variables supporting the idea that stomata can only exert short-term control of BVOC emissions (Niinemets and Reichstein, 2003; Schollert *et al.*, 2015).

### *Increased nutrient availability modifies* C. tetragona *anatomy responses caused by shading*

Treatments combining increased nutrient availability and shading had no effects on BVOC emissions. This is in contrast to results of short-term experiments combining increased nutrient availability with shading (Hartley *et al.*, 1995; Litvak *et al.*, 1996) but may have been constrained by factors such as natural variation and long-term acclimation of plants to the simulated environmental conditions. Increased nutrient availability modified some leaf anatomical responses caused by shading. For *C. tetragona*, the density of glandular trichomes facing the stem was reduced by increased nutrient availability in the shading treatments. In conditions with low light and increased nutrient availability, plants have a low carbon/nutrient ratio, and thus tend to allocate less carbon to the synthesis of secondary defence structures such as trichomes. This suggests that increased nutrient availability due to fertilization may have enhanced the effects of shading through its direct effect on the carbon/nutrient ratio. Increased intercellular space in the spongy tissue under shading in the absence of increased nutrient availability for *E. hermaphroditum* suggests a strategy to enhance photosynthesis by increasing internal light scattering and absorbance (DeLucia *et al*., 1996). The fact that the increase only occurred in the absence of fertilization may imply that higher soil nutrient availability somehow mitigates the effects of low light levels on photosynthetic capacity, presumably by promoting electron transport rate and CO_2_ fixation, and allowing an increase in leaf area which reduces the loss of photosynthate. In addition, fertilized plants under shaded conditions may experience lower mesophyll resistance, thereby enhancing their carbon gain capacity despite reduced light levels (Hartley *et al.*, 1995).

### Conclusions

In summary, increased nutrient availability has the potential to affect subarctic vegetation in the future by altering BVOC emissions and leaf anatomy in a time perspective of decades. Changes in both deciduous and evergreen shrubs are relevant, as both functional groups have been shown to increase with the ongoing climate warming, although in different areas (Vowles and Björk, 2019; Myers-Smith *et al.*, 2020). Our results provide new evidence of subarctic shrub responses, especially leaf anatomy, to increased nutrient availability under future conditions of climate warming and increased cloudiness. In terms of BVOCs, the deciduous *B. nana* was the most responsive plant species and monoterpenes the most responsive BVOC group, while the studied species showed contrasting responses in leaf anatomy. Notably, the density of glandular trichomes of *C. tetragona* was increased by fertilization under warming, with an opposite effect under shading. Glandular trichomes have distinct roles in plants: protection against herbivores, fungal and microbial diseases, and extreme temperatures (Valkama *et al.*, 2004; Glas *et al.*, 2012; Muravnik and Shavarda, 2012). Our findings suggest that as nutrient availability increases under future climatic conditions, subarctic shrubs will develop increased resistance to biotic and abiotic stresses under rising temperature as opposed to under increased cloudiness, which could lead to decreased resistance. More functional (intact) glandular trichomes resulting from increased nutrient availability, as was observed for *E. hermaphroditum*, could also enhance their defence capacity against biotic stress towards the end of the growth season. Our findings highlight the importance of changes in nutrient availability in the Subarctic, which can interact with the effects of climate warming and increased cloudiness.

## SUPPLEMENTARY DATA

Supplementary data are available at *Annals of Botany* online and consist of the following. Table S1: emissions of isoprene, non-oxygenated monoterpenes, oxygenated monoterpenes, sesquiterpenes, green-leaf volatiles and benzenoids from *E. hermaphroditum*, *C. tetragona* and *B. nana* under long-term control, shading, warming, fertilization, fertilization + shading and fertilization + warming treatments. Table S2: emissions of individual compounds from *E. hermaphroditum* under long-term control, shading and warming treatments. Table S3: emissions of individual compounds from *E. hermaphroditum* under long-term fertilization, fertilization + shading and fertilization + warming treatments. Table S4: emissions of individual compounds from *C. tetragona* under long-term control, shading and warming treatments. Table S5: emissions of individual compounds from *C. teragona* under long-term fertilization, fertilization + shading and fertilization + warming treatments. Table S6: emissions of individual compounds from *B. nana* under long-term control, shading and warming treatments. Table S7: emissions of individual compounds from *B. nana* under long-term fertilization, fertilization + shading and fertilization + warming treatments. Figure S1: cross-section of *Empetrum hermaphroditum* leaf from fertilization treatment. Figure S2: examples of different condition classes of *Empetrum hermaphroditum* glandular trichomes. Figure S3: percentage contributions of isoprene, green-leaf volatiles, monoterpenes, oxygenated monoterpenes, sesquiterpenes and benzenoids to total BVOC emissions from *E. hermaphroditum*, *C. tetragona* and *B. nana* averaged across treatments. Figure S4: PCA of the emissions of individual BVOCs from *E. hermaphroditum*. Figure S5: PCA of the emissions of individual BVOCs from *C. tetragona*.

mcac004_suppl_Supplementary_Figure_S1

mcac004_suppl_Supplementary_Figure_S2

mcac004_suppl_Supplementary_Figure_S3

mcac004_suppl_Supplementary_Figure_S4

mcac004_suppl_Supplementary_Figure_S5

mcac004_suppl_Supplementary_Table_S1

mcac004_suppl_Supplementary_Table_S2

mcac004_suppl_Supplementary_Table_S3

mcac004_suppl_Supplementary_Table_S4

mcac004_suppl_Supplementary_Table_S5

mcac004_suppl_Supplementary_Table_S6

mcac004_suppl_Supplementary_Table_S7
