# Supplementary figures and images for: Influence of increased nutrient availability on biogenic volatile organic compound (BVOC) emissions and leaf anatomy of subarctic dwarf shrubs under climate warming and increased cloudiness
Source: Ann Bot. 2022 Jan 13;129(4):443–55. doi: 10.1093/aob/mcac004 (PMC8944702; doi:10.1093/aob/mcac004)

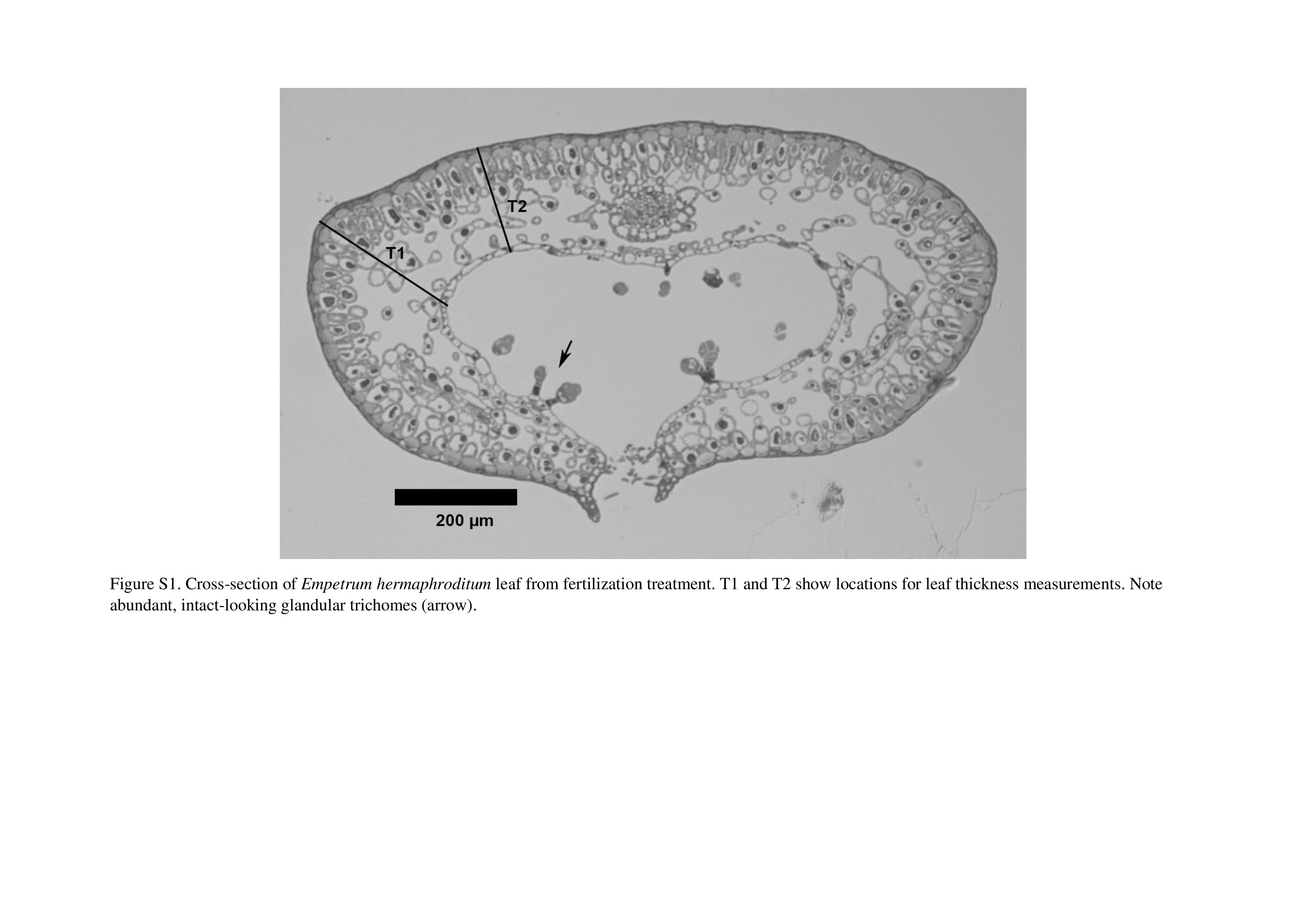

Supplement: mcac004_suppl_Supplementary_Figure_S1 [file mcac004_suppl_supplementary_figure_s1.jpeg]

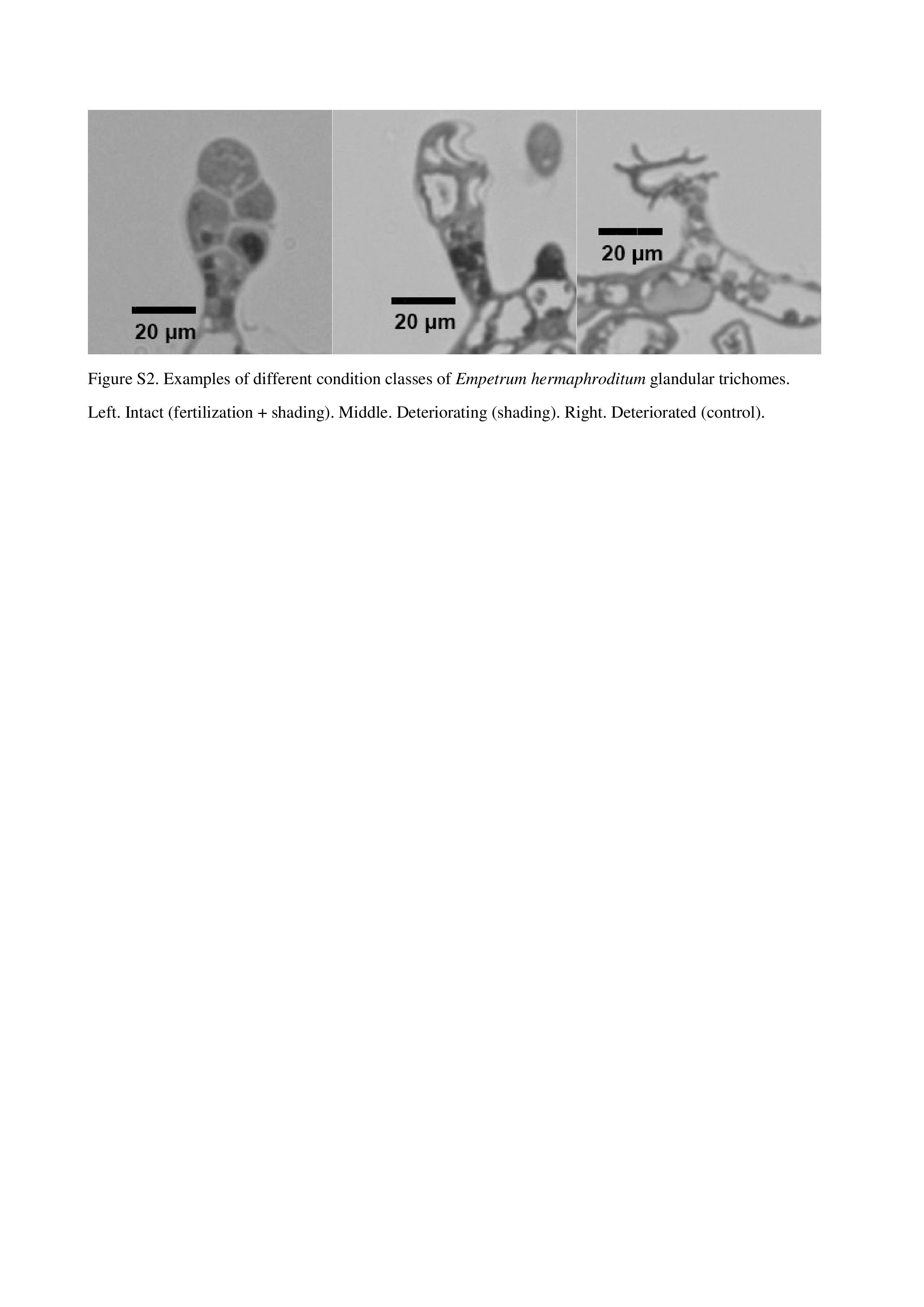

Supplement: mcac004_suppl_Supplementary_Figure_S2 [file mcac004_suppl_supplementary_figure_s2.jpeg]

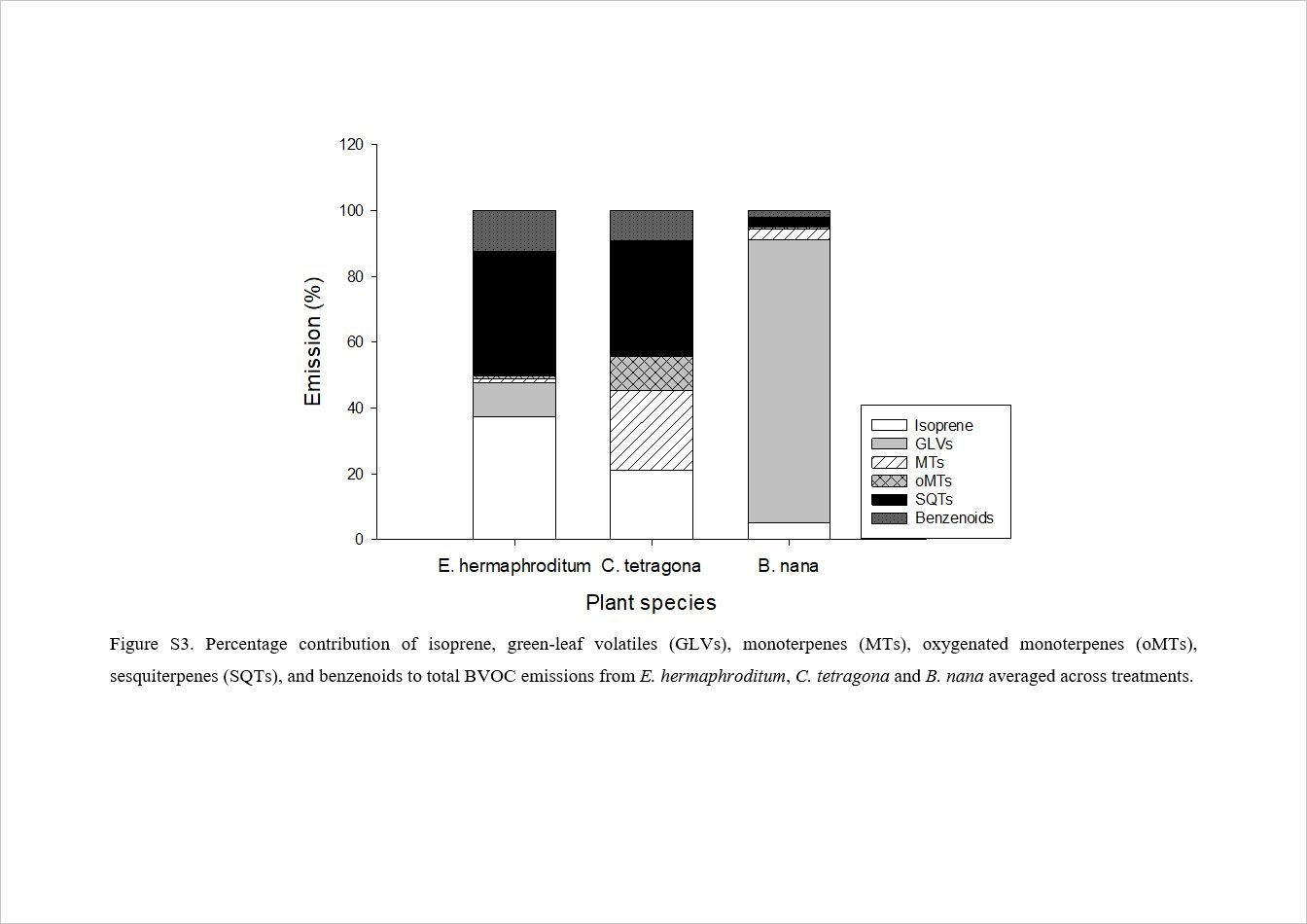

Supplement: mcac004_suppl_Supplementary_Figure_S3 [file mcac004_suppl_supplementary_figure_s3.jpeg]

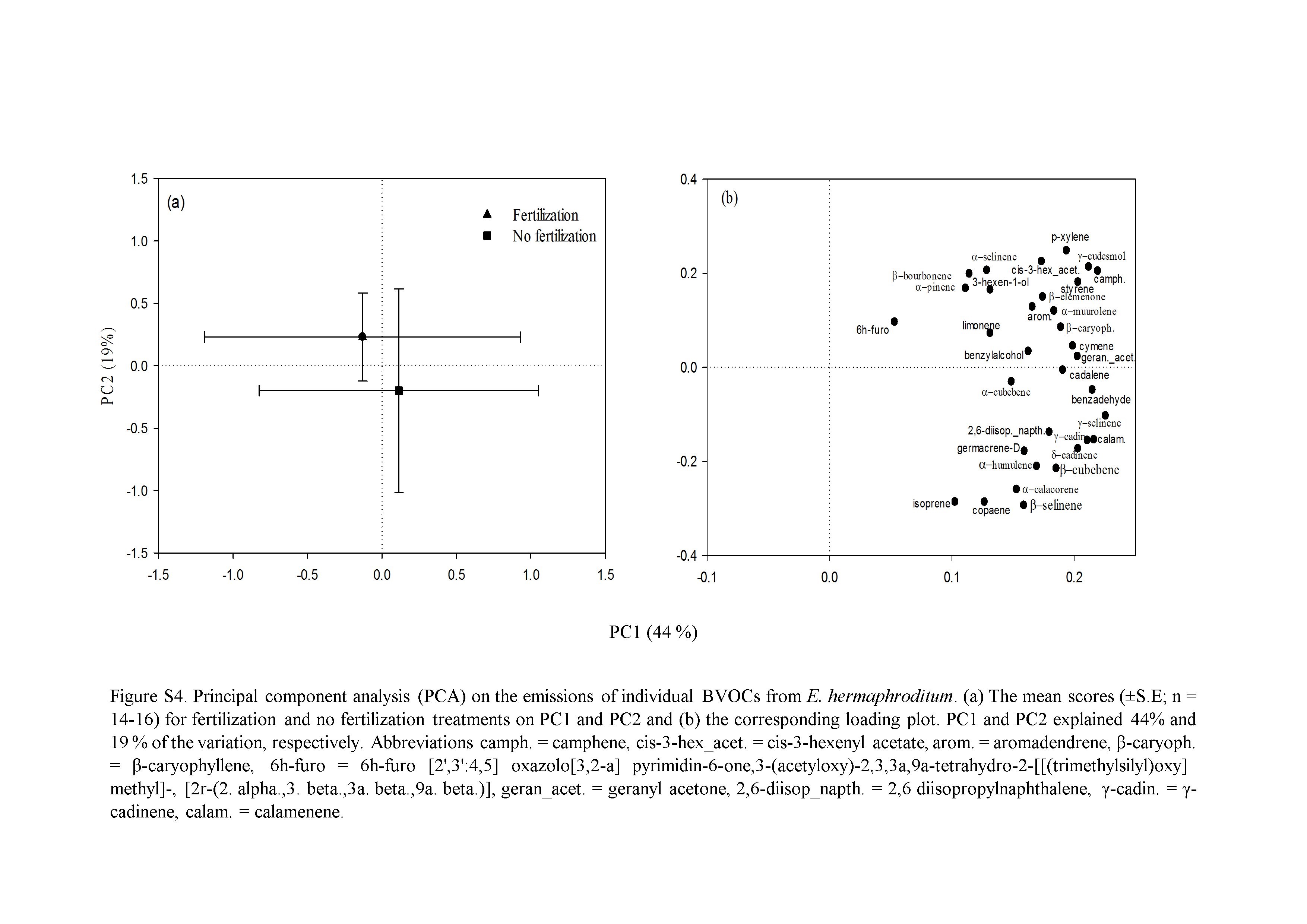

Supplement: mcac004_suppl_Supplementary_Figure_S4 [file mcac004_suppl_supplementary_figure_s4.jpeg]

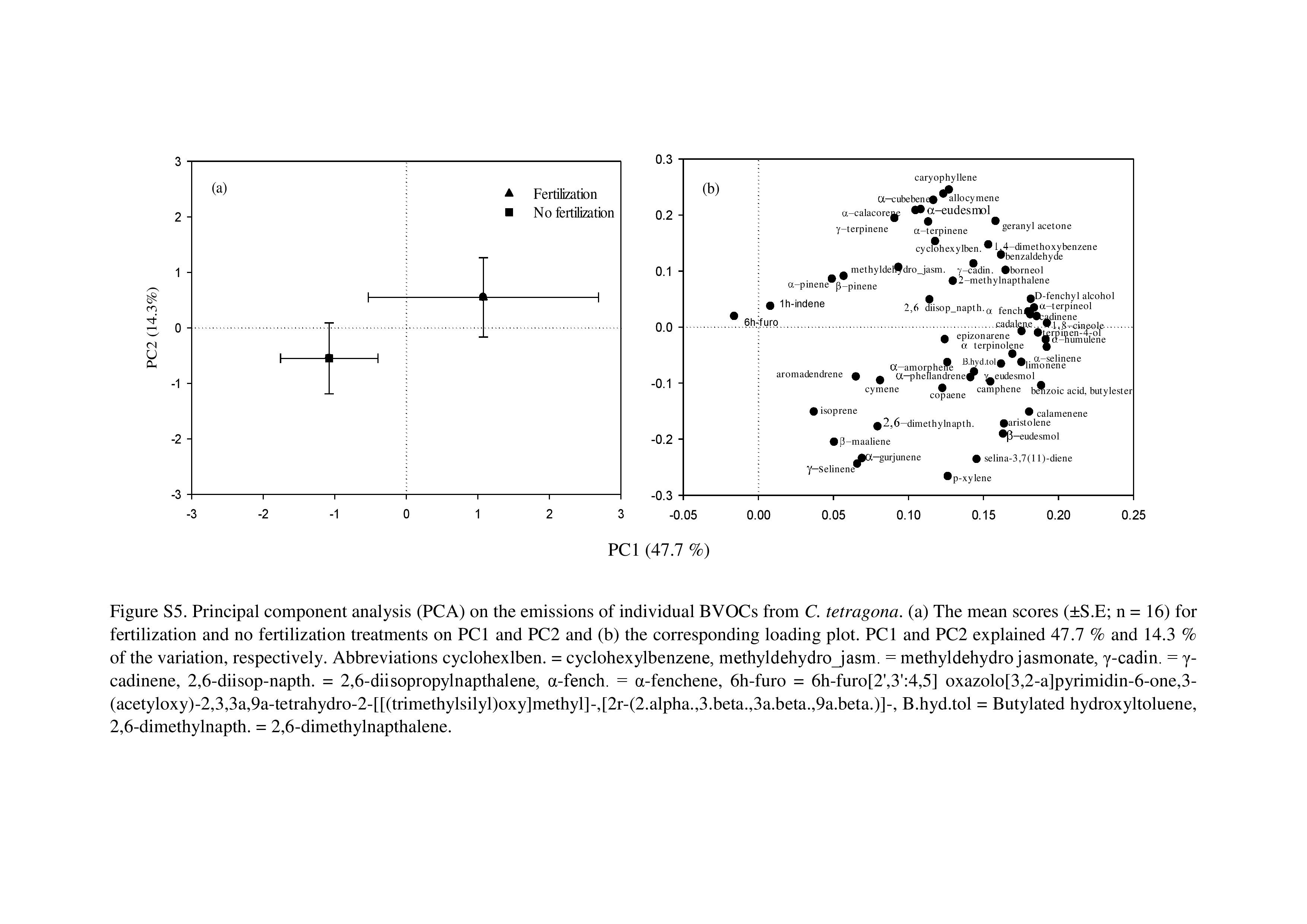

Supplement: mcac004_suppl_Supplementary_Figure_S5 [file mcac004_suppl_supplementary_figure_s5.jpeg]
