# Supplementary material for: Influence of increased nutrient availability on biogenic volatile organic compound (BVOC) emissions and leaf anatomy of subarctic dwarf shrubs under climate warming and increased cloudiness
Source: Ann Bot. 2022 Jan 13;129(4):443–55. doi: 10.1093/aob/mcac004 (PMC8944702; doi:10.1093/aob/mcac004)
Supplement: mcac004_suppl_Supplementary_Table_S1 [file mcac004_suppl_supplementary_table_s1.docx]

| Table S1. Emissions (µg g^-1^ h^-1^, mean ± SE, n = 5-6) of isoprene, non-oxygenated monoterpenes, oxygenated monoterpenes, sesquiterpenes, green-leaf volatiles, and benzenoids from *E. hermaphroditum, C. tetragona* and *B. nana* under long-term control (C), shading (S), warming (W), fertilization (F), fertilization + shading (FS) and fertilization + warming (FW) treatments. T = Climate Treatment, Fert = Fertilization, T × Fert = Climate Treatment × Fertilization. | | | | | | | | | |
| --- | --- | --- | --- | --- | --- | --- | --- | --- | --- |
|  |  |  |  |  |  |  |  | p-value |  |
|  | C | S | W | F | FS | FW | T | Fert | T × Fert |
| *E. hermaphroditum* |  |  |  |  |  |  |  |  |  |
| Isoprene | 0.1 ± 0.1 | 0.7 ± 0.5 | 1.0 ± 1.0 | 0.4 ± 0.4 | 0.0 ± 0.0 | 0.0 ± 0.0 | 0.921 | 0.240 | 0.303 |
| Non-Oxygenated Monoterpenes | 0.0 ± 0.0 | 0.0 ± 0.0 | 0.0 ± 0.0 | 0.0 ± 0.0 | 0.0 ± 0.0 | 0.0 ± 0.0 | 0.596 | 0.811 | 0.992 |
| Oxygenated Monoterpenes | 0.0 ± 0.0 | 0.2 ± 0.1 | 0.0 ± 0.0 | 0.0 ± 0.0 | 0.1 ± 0.1 | 0.0 ± 0.0 | **0.044** | 0.501 | 0.624 |
| Sesquiterpenes | 0.2 ± 0.1 | 1.0 ± 0.6 | 0.1 ± 0.0 | 0.6 ± 0.5 | 0.2 ± 0.1 | 0.2 ± 0.2 | 0.517 | 0.555 | 0.321 |
| Green-leaf volatiles | 0.1 ± 0.1 | 0.2 ± 0.2 | 0.2 ± 0.2 | 0.1 ± 0.1 | 0.1 ± 0.1 | 0.0 ± 0.0 | 0.805 | 0.350 | 0.769 |
| Benzenoids | 0.1 ± 0.1 | 0.2 ± 0.1 | 0.0 ± 0.0 | 0.2 ± 0.2 | 0.2 ± 0.2 | 0.0 ± 0.0 | 0.426 | 0.757 | 0.952 |
| *C. tetragona* |  |  |  |  |  |  |  |  |  |
| Isoprene | 0.6 ± 0.6 | 0.6 ± 0.5 | 3.3 ± 1.7 | 0.2 ± 0.1 | 0.2 ± 0.2 | 0.4 ± 0.4 | 0.157 | **0.051** | 0.260 |
| Non-Oxygenated Monoterpenes | 0.0 ± 0.0 | 0.3 ± 0.2 | 0.2 ± 0.1 | 3.7 ± 3.7 | 0.5 ± 0.2 | 0.7 ± 0.5 | 0.966 | 0.125 | 0.647 |
| Oxygenated Monoterpenes | 0.0 ± 0.0 | 0.1 ± 0.0 | 0.0 ± 0.0 | 1.5 ± 1.5 | 0.3 ± 0.2 | 0.4 ± 0.3 | 0.948 | 0.107 | 0.781 |
| Sesquiterpenes | 0.0 ± 0.0 | 0.1 ± 0.1 | 0.8 ± 0.8 | 5.3 ± 5.3 | 0.5 ± 0.3 | 1.1 ± 0.8 | 0.851 | 0.139 | 0.716 |
| Benzenoids | 0.0 ± 0.0 | 0.3 ± 0.2 | 0.2 ± 0.2 | 0.8 ± 0.8 | 0.4 ± 0.2 | 0.4 ± 0.3 | 0.829 | 0.203 | 0.750 |
| *B. Nana* |  |  |  |  |  |  |  |  |  |
| Isoprene | 0.2 ± 0.1 | 0.3 ± 0.3 | 0.4 ± 0.3 | 0.0 ± 0.0 | 1.5 ± 1.4 | 0.0 ± 0.0 | 0.639 | 0.682 | 0.665 |
| Non-Oxygenated Monoterpenes | 0.1 ± 0.1 | 0.0 ± 0.0 | 0.1 ± 0.1 | 0.4 ± 0.3 | 0.5 ± 0.2 | 0.5 ± 0.2 | 0.909 | **0.009** | 0.826 |
| Oxygenated Monoterpenes | 0.0 ± 0.0 | 0.1 ± 0.1 | 0.1 ± 0.1 | 0.1 ± 0.0 | 0.0 ± 0.0 | 0.0 ± 0.0 | 0.937 | 0.837 | 0.489 |
| Sesquiterpenes | 0.1 ± 0.1 | 0.3 ± 0.2 | 0.5 ± 0.5 | 0.4 ± 0.3 | 0.3 ± 0.2 | 0.1 ± 0.1 | 0.936 | 0.982 | 0.511 |
| Green-leaf volatiles | 7.2 ± 6.9 | 19.1 ± 18.8 | 9.7 ± 9.0 | 10.7 ± 10.2 | 2.5 ± 1.3 | 1.7 ± 1.2 | 0.753 | 0.654 | 0.467 |
| Benzenoids | 0.3 ± 0.3 | 0.1 ± 0.1 | 0.2 ± 0.2 | 0.4 ± 0.4 | 0.0 ± 0.0 | 0.0 ± 0.0 | 0.277 | 0.734 | 0.539 |

P-values from LMM ANOVA and p<0.1 emboldened.
