# Supplementary material for: Influence of increased nutrient availability on biogenic volatile organic compound (BVOC) emissions and leaf anatomy of subarctic dwarf shrubs under climate warming and increased cloudiness
Source: Ann Bot. 2022 Jan 13;129(4):443–55. doi: 10.1093/aob/mcac004 (PMC8944702; doi:10.1093/aob/mcac004)
Supplement: mcac004_suppl_Supplementary_Table_S2 [file mcac004_suppl_supplementary_table_s2.docx]

Table S2. Emissions (µg g^-1^ h^-1^) of individual compounds from *E. hermaphroditum* under long-term control (C), shading (S), and warming (W) treatments.

|  | Control (C) | | | | | | Shading (S) | | | | | Warming (W) | | | | |
| --- | --- | --- | --- | --- | --- | --- | --- | --- | --- | --- | --- | --- | --- | --- | --- | --- |
|  | C | C | C | C | C | C | S | S | S | S | S | W | W | W | W | W |
| isoprene | 0.09 | 0.31 | 0.00 | 0.00 | 0.00 | 0.00 | 0.29 | 0.00 | 2.73 | 0.22 | 0.28 | 4.79 | 0.10 | 0.00 | 0.00 | 0.00 |
| cis-3-hexen-1-ol | 0.00 | 0.00 | 0.00 | 0.00 | 0.00 | 0.00 | 0.00 | 0.38 | 0.00 | 0.00 | 0.00 | 0.21 | 0.00 | 0.00 | 0.00 | 0.00 |
| cis-3-hexenyl acetate | 0.02 | 0.00 | 0.00 | 0.44 | 0.00 | 0.00 | 0.00 | 0.53 | 0.00 | 0.00 | 0.00 | 0.69 | 0.00 | 0.00 | 0.00 | 0.00 |
| α-pinene | 0.00 | 0.00 | 0.00 | 0.03 | 0.00 | 0.00 | 0.00 | 0.00 | 0.00 | 0.00 | 0.00 | 0.00 | 0.00 | 0.00 | 0.00 | 0.00 |
| camphene | 0.00 | 0.00 | 0.00 | 0.02 | 0.00 | 0.00 | 0.00 | 0.02 | 0.00 | 0.00 | 0.00 | 0.00 | 0.00 | 0.00 | 0.00 | 0.00 |
| cymene | 0.00 | 0.00 | 0.00 | 0.05 | 0.00 | 0.00 | 0.00 | 0.03 | 0.02 | 0.00 | 0.00 | 0.00 | 0.00 | 0.00 | 0.00 | 0.00 |
| limonene | 0.00 | 0.00 | 0.00 | 0.00 | 0.00 | 0.00 | 0.00 | 0.00 | 0.01 | 0.00 | 0.00 | 0.00 | 0.00 | 0.00 | 0.00 | 0.00 |
| geranyl acetone | 0.00 | 0.00 | 0.00 | 0.00 | 0.00 | 0.00 | 0.00 | 0.09 | 0.08 | 0.00 | 0.00 | 0.00 | 0.00 | 0.00 | 0.00 | 0.00 |
| α-cubebene | 0.01 | 0.00 | 0.00 | 0.08 | 0.05 | 0.03 | 0.02 | 0.02 | 0.14 | 0.01 | 0.02 | 0.00 | 0.00 | 0.02 | 0.00 | 0.01 |
| copaene | 0.05 | 0.04 | 0.00 | 0.00 | 0.00 | 0.24 | 0.14 | 0.13 | 0.90 | 0.00 | 0.14 | 0.05 | 0.03 | 0.13 | 0.00 | 0.06 |
| β-bourbonene | 0.00 | 0.00 | 0.01 | 0.06 | 0.01 | 0.00 | 0.00 | 0.04 | 0.00 | 0.00 | 0.00 | 0.00 | 0.00 | 0.00 | 0.00 | 0.00 |
| β-cubebene | 0.00 | 0.00 | 0.00 | 0.00 | 0.00 | 0.00 | 0.00 | 0.00 | 0.05 | 0.00 | 0.00 | 0.02 | 0.00 | 0.01 | 0.00 | 0.00 |
| β-caryophyllene | 0.10 | 0.00 | 0.00 | 0.33 | 0.00 | 0.00 | 0.00 | 0.35 | 0.18 | 0.00 | 0.01 | 0.07 | 0.01 | 0.00 | 0.00 | 0.00 |
| α-humulene | 0.00 | 0.00 | 0.00 | 0.06 | 0.00 | 0.00 | 0.00 | 0.07 | 0.20 | 0.00 | 0.00 | 0.00 | 0.00 | 0.00 | 0.00 | 0.00 |
| germacrene-D | 0.00 | 0.00 | 0.00 | 0.00 | 0.00 | 0.00 | 0.02 | 0.00 | 0.05 | 0.00 | 0.00 | 0.01 | 0.00 | 0.00 | 0.00 | 0.01 |
| β-selinene | 0.00 | 0.00 | 0.00 | 0.00 | 0.00 | 0.00 | 0.00 | 0.05 | 0.99 | 0.00 | 0.00 | 0.00 | 0.00 | 0.00 | 0.00 | 0.00 |
| α-Muurolene | 0.00 | 0.00 | 0.00 | 0.01 | 0.00 | 0.00 | 0.00 | 0.02 | 0.00 | 0.00 | 0.00 | 0.00 | 0.00 | 0.00 | 0.00 | 0.00 |
| α-selinene | 0.00 | 0.00 | 0.00 | 0.01 | 0.00 | 0.00 | 0.00 | 0.29 | 0.00 | 0.00 | 0.00 | 0.00 | 0.00 | 0.00 | 0.00 | 0.00 |
| γ-cadinene | 0.00 | 0.00 | 0.00 | 0.00 | 0.00 | 0.00 | 0.00 | 0.03 | 0.10 | 0.00 | 0.00 | 0.00 | 0.00 | 0.00 | 0.00 | 0.00 |
| δ-cadinene | 0.01 | 0.00 | 0.00 | 0.03 | 0.01 | 0.04 | 0.02 | 0.03 | 0.25 | 0.00 | 0.03 | 0.00 | 0.00 | 0.02 | 0.00 | 0.00 |
| calamenene | 0.00 | 0.00 | 0.00 | 0.05 | 0.00 | 0.00 | 0.00 | 0.02 | 0.17 | 0.00 | 0.00 | 0.00 | 0.00 | 0.00 | 0.00 | 0.00 |
| α-calacorene | 0.00 | 0.00 | 0.00 | 0.02 | 0.00 | 0.00 | 0.00 | 0.01 | 0.05 | 0.00 | 0.00 | 0.00 | 0.00 | 0.00 | 0.00 | 0.00 |
| β-elemenone | 0.00 | 0.00 | 0.00 | 0.06 | 0.00 | 0.00 | 0.00 | 0.00 | 0.00 | 0.00 | 0.00 | 0.00 | 0.00 | 0.00 | 0.00 | 0.00 |
| aromadendrene | 0.00 | 0.00 | 0.00 | 0.00 | 0.00 | 0.00 | 0.00 | 0.03 | 0.00 | 0.00 | 0.00 | 0.00 | 0.00 | 0.02 | 0.00 | 0.00 |
| γ-eudesmol | 0.00 | 0.00 | 0.00 | 0.05 | 0.00 | 0.00 | 0.00 | 0.13 | 0.00 | 0.00 | 0.00 | 0.00 | 0.00 | 0.00 | 0.00 | 0.00 |
| γ-selinene | 0.00 | 0.00 | 0.00 | 0.03 | 0.00 | 0.00 | 0.00 | 0.04 | 0.08 | 0.00 | 0.00 | 0.00 | 0.00 | 0.00 | 0.00 | 0.00 |
| cadalene | 0.00 | 0.00 | 0.00 | 0.11 | 0.00 | 0.00 | 0.00 | 0.00 | 0.09 | 0.00 | 0.00 | 0.00 | 0.00 | 0.00 | 0.00 | 0.00 |
| styrene | 0.00 | 0.00 | 0.00 | 0.05 | 0.00 | 0.00 | 0.00 | 0.08 | 0.00 | 0.00 | 0.00 | 0.00 | 0.00 | 0.00 | 0.00 | 0.00 |
| p-xylene | 0.00 | 0.00 | 0.00 | 0.10 | 0.00 | 0.00 | 0.01 | 0.19 | 0.00 | 0.00 | 0.00 | 0.01 | 0.00 | 0.00 | 0.00 | 0.00 |
| benzaldehyde | 0.00 | 0.00 | 0.00 | 0.00 | 0.00 | 0.00 | 0.00 | 0.00 | 0.47 | 0.00 | 0.00 | 0.00 | 0.00 | 0.00 | 0.00 | 0.00 |
| benzylalcohol | 0.00 | 0.00 | 0.00 | 0.06 | 0.00 | 0.00 | 0.00 | 0.00 | 0.06 | 0.00 | 0.00 | 0.00 | 0.00 | 0.00 | 0.00 | 0.00 |
| 6h-furo [2',3':4,5]oxazolo [3,2-a]pyrimidin  -6-one, 3-(acetyloxy)-2,3,3a,9a-tetrahydro-2-  [[(trimethylsilyl)oxy]methyl]- | 0.00 | 0.00 | 0.01 | 0.28 | 0.00 | 0.00 | 0.00 | 0.00 | 0.00 | 0.00 | 0.00 | 0.00 | 0.00 | 0.00 | 0.00 | 0.00 |
| 2,6-diisopropylnaphthalene | 0.00 | 0.00 | 0.00 | 0.03 | 0.00 | 0.00 | 0.00 | 0.00 | 0.09 | 0.00 | 0.00 | 0.00 | 0.00 | 0.00 | 0.00 | 0.00 |
