# Supplementary material for: Influence of increased nutrient availability on biogenic volatile organic compound (BVOC) emissions and leaf anatomy of subarctic dwarf shrubs under climate warming and increased cloudiness
Source: Ann Bot. 2022 Jan 13;129(4):443–55. doi: 10.1093/aob/mcac004 (PMC8944702; doi:10.1093/aob/mcac004)
Supplement: mcac004_suppl_Supplementary_Table_S4 [file mcac004_suppl_supplementary_table_s4.docx]

Table S4. Emissions (µg g^-1^ h^-1^) of individual compounds from *C. tetragona* under long-term control (C), shading (S) and warming (W) treatments.

|  | Control (C) | | | | | | Shading (S) | | | | | Warming (W) | | | | |
| --- | --- | --- | --- | --- | --- | --- | --- | --- | --- | --- | --- | --- | --- | --- | --- | --- |
|  | C | C | C | C | C | C | S | S | S | S | S | W | W | W | W | W |
| isoprene | 0.07 | 3.60 | 0.00 | 0.00 | 0.00 | 0.00 | 0.00 | 0.36 | 0.00 | 2.58 | 0.00 | 8.12 | 6.77 | 1.79 | 0.00 | 0.00 |
| α-pinene | 0.00 | 0.00 | 0.00 | 0.00 | 0.01 | 0.00 | 0.00 | 0.00 | 0.01 | 0.16 | 0.01 | 0.00 | 0.00 | 0.01 | 0.00 | 0.00 |
| α-fenchene | 0.00 | 0.00 | 0.00 | 0.00 | 0.00 | 0.00 | 0.01 | 0.00 | 0.00 | 0.00 | 0.00 | 0.00 | 0.00 | 0.00 | 0.00 | 0.00 |
| camphene | 0.00 | 0.00 | 0.00 | 0.00 | 0.00 | 0.00 | 0.06 | 0.01 | 0.00 | 0.05 | 0.00 | 0.03 | 0.00 | 0.00 | 0.00 | 0.00 |
| β-pinene | 0.00 | 0.00 | 0.00 | 0.00 | 0.00 | 0.00 | 0.00 | 0.00 | 0.00 | 0.12 | 0.00 | 0.00 | 0.00 | 0.00 | 0.00 | 0.00 |
| α-terpinene | 0.01 | 0.01 | 0.00 | 0.00 | 0.00 | 0.00 | 0.00 | 0.00 | 0.00 | 0.08 | 0.00 | 0.04 | 0.01 | 0.00 | 0.00 | 0.00 |
| cymene | 0.00 | 0.01 | 0.00 | 0.00 | 0.01 | 0.01 | 0.22 | 0.02 | 0.00 | 0.18 | 0.00 | 0.46 | 0.01 | 0.00 | 0.00 | 0.00 |
| limonene | 0.01 | 0.00 | 0.00 | 0.00 | 0.01 | 0.00 | 0.31 | 0.05 | 0.00 | 0.15 | 0.00 | 0.16 | 0.01 | 0.00 | 0.00 | 0.00 |
| α-phellandrene | 0.00 | 0.00 | 0.00 | 0.00 | 0.00 | 0.00 | 0.00 | 0.01 | 0.00 | 0.00 | 0.00 | 0.00 | 0.00 | 0.00 | 0.00 | 0.00 |
| γ-terpinene | 0.01 | 0.00 | 0.00 | 0.00 | 0.00 | 0.00 | 0.00 | 0.00 | 0.00 | 0.00 | 0.00 | 0.00 | 0.00 | 0.00 | 0.00 | 0.00 |
| α-terpinolene | 0.00 | 0.00 | 0.00 | 0.00 | 0.00 | 0.00 | 0.08 | 0.01 | 0.00 | 0.07 | 0.00 | 0.00 | 0.00 | 0.00 | 0.00 | 0.00 |
| 1,8-cineole | 0.00 | 0.01 | 0.00 | 0.00 | 0.01 | 0.02 | 0.11 | 0.04 | 0.00 | 0.10 | 0.01 | 0.12 | 0.01 | 0.00 | 0.00 | 0.00 |
| D-fenchyl alcohol | 0.00 | 0.00 | 0.00 | 0.00 | 0.00 | 0.00 | 0.01 | 0.00 | 0.00 | 0.00 | 0.00 | 0.00 | 0.00 | 0.00 | 0.00 | 0.00 |
| borneol | 0.00 | 0.00 | 0.00 | 0.00 | 0.00 | 0.00 | 0.03 | 0.00 | 0.00 | 0.00 | 0.00 | 0.00 | 0.00 | 0.00 | 0.00 | 0.00 |
| terpinen-4-ol | 0.00 | 0.00 | 0.00 | 0.00 | 0.00 | 0.00 | 0.00 | 0.00 | 0.00 | 0.00 | 0.00 | 0.06 | 0.00 | 0.00 | 0.00 | 0.00 |
| α-terpineol | 0.00 | 0.00 | 0.00 | 0.00 | 0.00 | 0.00 | 0.00 | 0.02 | 0.00 | 0.10 | 0.00 | 0.00 | 0.00 | 0.00 | 0.00 | 0.00 |
| geranyl acetone | 0.00 | 0.00 | 0.00 | 0.00 | 0.00 | 0.00 | 0.04 | 0.00 | 0.00 | 0.01 | 0.00 | 0.00 | 0.00 | 0.00 | 0.00 | 0.00 |
| methyl dehydrojasmonate | 0.00 | 0.00 | 0.00 | 0.00 | 0.00 | 0.00 | 0.00 | 0.00 | 0.04 | 0.00 | 0.00 | 0.05 | 0.00 | 0.00 | 0.00 | 0.00 |
| α-cubebene | 0.00 | 0.00 | 0.00 | 0.00 | 0.00 | 0.01 | 0.00 | 0.02 | 0.00 | 0.07 | 0.00 | 0.00 | 0.00 | 0.00 | 0.00 | 0.00 |
| copaene | 0.00 | 0.00 | 0.00 | 0.00 | 0.00 | 0.00 | 0.07 | 0.00 | 0.00 | 0.00 | 0.00 | 0.02 | 0.00 | 0.00 | 0.00 | 0.00 |
| caryophyllene | 0.00 | 0.00 | 0.00 | 0.00 | 0.00 | 0.00 | 0.01 | 0.00 | 0.00 | 0.00 | 0.00 | 0.00 | 0.00 | 0.00 | 0.00 | 0.00 |
| α-humulene | 0.00 | 0.00 | 0.00 | 0.00 | 0.00 | 0.00 | 0.02 | 0.00 | 0.00 | 0.00 | 0.00 | 0.07 | 0.00 | 0.00 | 0.00 | 0.00 |
| γ-cadinene | 0.00 | 0.00 | 0.00 | 0.00 | 0.00 | 0.00 | 0.04 | 0.00 | 0.00 | 0.02 | 0.00 | 0.03 | 0.00 | 0.00 | 0.00 | 0.00 |
| β-selinene | 0.00 | 0.00 | 0.00 | 0.00 | 0.00 | 0.00 | 0.08 | 0.00 | 0.00 | 0.00 | 0.00 | 0.50 | 0.00 | 0.00 | 0.00 | 0.00 |
| α-selinene | 0.00 | 0.00 | 0.00 | 0.00 | 0.00 | 0.00 | 0.07 | 0.00 | 0.00 | 0.04 | 0.00 | 0.17 | 0.00 | 0.00 | 0.00 | 0.00 |
| α-amorphene | 0.00 | 0.00 | 0.00 | 0.00 | 0.00 | 0.00 | 0.00 | 0.02 | 0.00 | 0.00 | 0.00 | 0.00 | 0.00 | 0.00 | 0.00 | 0.00 |
| cadinene | 0.00 | 0.00 | 0.00 | 0.00 | 0.01 | 0.01 | 0.07 | 0.03 | 0.00 | 0.00 | 0.01 | 0.07 | 0.01 | 0.00 | 0.00 | 0.00 |
| calamenene | 0.00 | 0.00 | 0.00 | 0.00 | 0.00 | 0.00 | 0.02 | 0.00 | 0.00 | 0.01 | 0.00 | 0.16 | 0.00 | 0.00 | 0.00 | 0.00 |
| aromadendrene | 0.00 | 0.00 | 0.00 | 0.00 | 0.00 | 0.00 | 0.00 | 0.00 | 0.00 | 0.00 | 0.00 | 0.01 | 0.00 | 0.00 | 0.00 | 0.00 |
| α-gurjunene | 0.00 | 0.00 | 0.00 | 0.00 | 0.00 | 0.00 | 0.00 | 0.00 | 0.00 | 0.00 | 0.00 | 0.10 | 0.00 | 0.00 | 0.00 | 0.00 |
| α-calacorene | 0.00 | 0.00 | 0.00 | 0.00 | 0.00 | 0.00 | 0.02 | 0.00 | 0.00 | 0.00 | 0.00 | 0.00 | 0.00 | 0.00 | 0.00 | 0.00 |
| selina-3,7(11)-diene | 0.00 | 0.00 | 0.00 | 0.00 | 0.00 | 0.00 | 0.00 | 0.00 | 0.00 | 0.00 | 0.00 | 0.15 | 0.00 | 0.00 | 0.00 | 0.00 |
| γ-selinene | 0.00 | 0.00 | 0.00 | 0.00 | 0.01 | 0.00 | 0.00 | 0.00 | 0.00 | 0.00 | 0.00 | 0.99 | 0.00 | 0.00 | 0.00 | 0.00 |
| aristolene | 0.00 | 0.00 | 0.00 | 0.00 | 0.00 | 0.00 | 0.00 | 0.01 | 0.00 | 0.00 | 0.00 | 0.13 | 0.00 | 0.00 | 0.00 | 0.00 |
| β-eudesmol | 0.00 | 0.00 | 0.00 | 0.00 | 0.00 | 0.00 | 0.00 | 0.00 | 0.00 | 0.00 | 0.00 | 0.93 | 0.00 | 0.00 | 0.00 | 0.00 |
| β-maaliene | 0.00 | 0.00 | 0.00 | 0.00 | 0.00 | 0.00 | 0.02 | 0.00 | 0.00 | 0.00 | 0.00 | 0.54 | 0.00 | 0.00 | 0.00 | 0.00 |
| γ-eudesmol | 0.00 | 0.00 | 0.00 | 0.00 | 0.01 | 0.00 | 0.00 | 0.00 | 0.00 | 0.03 | 0.00 | 0.00 | 0.00 | 0.00 | 0.00 | 0.00 |
| α-eudesmol | 0.00 | 0.00 | 0.00 | 0.00 | 0.02 | 0.00 | 0.00 | 0.00 | 0.00 | 0.06 | 0.00 | 0.00 | 0.00 | 0.00 | 0.00 | 0.00 |
| cadalene | 0.00 | 0.00 | 0.00 | 0.00 | 0.00 | 0.00 | 0.00 | 0.00 | 0.00 | 0.00 | 0.00 | 0.10 | 0.00 | 0.00 | 0.00 | 0.00 |
| p-xylene | 0.00 | 0.00 | 0.00 | 0.00 | 0.00 | 0.00 | 0.00 | 0.00 | 0.00 | 0.04 | 0.00 | 0.11 | 0.00 | 0.00 | 0.00 | 0.00 |
| benzaldehyde | 0.00 | 0.00 | 0.00 | 0.00 | 0.00 | 0.00 | 0.00 | 0.00 | 0.00 | 0.56 | 0.00 | 0.00 | 0.00 | 0.00 | 0.00 | 0.00 |
| 1,4-dimethoxybenzene | 0.00 | 0.00 | 0.00 | 0.00 | 0.00 | 0.00 | 0.00 | 0.00 | 0.00 | 0.09 | 0.00 | 0.00 | 0.00 | 0.00 | 0.00 | 0.00 |
| 2-methylnaphthalene | 0.00 | 0.00 | 0.00 | 0.00 | 0.00 | 0.00 | 0.03 | 0.00 | 0.00 | 0.00 | 0.00 | 0.01 | 0.00 | 0.00 | 0.00 | 0.00 |
| 6h-furo [2',3':4,5]oxazolo [3,2-a]pyrimidin-6-one,  3-(acetyloxy)-2,3,3a,9a-tetrahydro-2-  [[(trimethylsilyl)oxy]methyl]- | 0.00 | 0.00 | 0.01 | 0.00 | 0.00 | 0.00 | 0.00 | 0.00 | 0.00 | 0.00 | 0.00 | 0.00 | 0.00 | 0.00 | 0.00 | 0.01 |
| cyclohexylbenzene | 0.00 | 0.00 | 0.00 | 0.00 | 0.00 | 0.00 | 0.09 | 0.00 | 0.00 | 0.00 | 0.00 | 0.00 | 0.00 | 0.00 | 0.00 | 0.00 |
| benzoicacid,butylester | 0.00 | 0.00 | 0.00 | 0.00 | 0.00 | 0.00 | 0.13 | 0.00 | 0.00 | 0.25 | 0.00 | 0.60 | 0.00 | 0.00 | 0.00 | 0.00 |
| 2,6-dimethylnaphthalene | 0.00 | 0.00 | 0.00 | 0.00 | 0.00 | 0.00 | 0.00 | 0.00 | 0.00 | 0.01 | 0.00 | 0.06 | 0.00 | 0.00 | 0.00 | 0.00 |
| butylated hydroxytoluene | 0.00 | 0.00 | 0.00 | 0.00 | 0.00 | 0.00 | 0.02 | 0.00 | 0.00 | 0.05 | 0.00 | 0.04 | 0.00 | 0.00 | 0.00 | 0.00 |
| 1h-indene | 0.00 | 0.00 | 0.00 | 0.00 | 0.00 | 0.00 | 0.11 | 0.00 | 0.00 | 0.00 | 0.00 | 0.00 | 0.00 | 0.00 | 0.00 | 0.00 |
| 2,6-diisopropylnaphthalene | 0.00 | 0.00 | 0.00 | 0.00 | 0.00 | 0.00 | 0.04 | 0.00 | 0.00 | 0.14 | 0.00 | 0.08 | 0.00 | 0.00 | 0.00 | 0.00 |
|  |  |  |  |  |  |  |  |  |  |  |  |  |  |  |  |  |
