# Supplementary material for: Influence of increased nutrient availability on biogenic volatile organic compound (BVOC) emissions and leaf anatomy of subarctic dwarf shrubs under climate warming and increased cloudiness
Source: Ann Bot. 2022 Jan 13;129(4):443–55. doi: 10.1093/aob/mcac004 (PMC8944702; doi:10.1093/aob/mcac004)
Supplement: mcac004_suppl_Supplementary_Table_S5 [file mcac004_suppl_supplementary_table_s5.docx]

Table S5. Emissions (µg g^-1^ h^-1^) of individual compounds from *C. teragona* under long-term fertilization (F), fertilization + shading (FS), and fertilization + warming (FW) treatments.

|  | Fertilization (F) | | | | | | Fertilization + Shading (FS) | | | | | | | Fertilization + Warming (FW) | | | | | |
| --- | --- | --- | --- | --- | --- | --- | --- | --- | --- | --- | --- | --- | --- | --- | --- | --- | --- | --- | --- |
|  | F | F | F | F | F | F | FS | FS | FS | FS | FS | FS | FW | | FW | FW | FW | FW |  |
| isoprene | 0.82 | 0.00 | 0.00 | 0.26 | 0.00 | 0.00 | 0.94 | 0.00 | 0.00 | 0.00 | 0.00 | 0.00 | 0.00 | | 1.78 | 0.00 | 0.00 | 0.00 |  |
| α-pinene | 0.00 | 0.00 | 0.01 | 0.00 | 0.02 | 0.00 | 0.00 | 0.03 | 0.15 | 0.00 | 0.00 | 0.00 | 0.00 | | 0.00 | 0.00 | 0.00 | 0.00 |  |
| α-fenchene | 0.00 | 0.00 | 0.00 | 0.00 | 0.00 | 0.00 | 0.00 | 0.00 | 0.01 | 0.01 | 0.00 | 0.00 | 0.00 | | 0.04 | 0.00 | 0.01 | 0.00 |  |
| camphene | 3.96 | 0.00 | 0.00 | 0.00 | 0.01 | 0.00 | 0.00 | 0.02 | 0.08 | 0.00 | 0.01 | 0.00 | 0.01 | | 0.22 | 0.00 | 0.04 | 0.00 |  |
| β-pinene | 0.00 | 0.00 | 0.00 | 0.00 | 0.00 | 0.00 | 0.00 | 0.00 | 0.11 | 0.00 | 0.00 | 0.00 | 0.00 | | 0.00 | 0.00 | 0.13 | 0.00 |  |
| α-terpinene | 0.04 | 0.00 | 0.00 | 0.00 | 0.00 | 0.00 | 0.00 | 0.14 | 0.09 | 0.16 | 0.01 | 0.01 | 0.00 | | 0.00 | 0.00 | 0.09 | 0.00 |  |
| cymene | 7.91 | 0.00 | 0.00 | 0.00 | 0.00 | 0.01 | 0.01 | 0.05 | 0.22 | 0.00 | 0.01 | 0.01 | 0.02 | | 0.00 | 0.00 | 0.24 | 0.01 |  |
| limonene | 6.65 | 0.00 | 0.00 | 0.00 | 0.01 | 0.00 | 0.01 | 0.14 | 0.24 | 0.34 | 0.01 | 0.00 | 0.04 | | 1.42 | 0.00 | 0.00 | 0.00 |  |
| α-phellandrene | 3.57 | 0.00 | 0.00 | 0.00 | 0.00 | 0.00 | 0.00 | 0.02 | 0.04 | 0.00 | 0.00 | 0.00 | 0.00 | | 0.17 | 0.00 | 0.05 | 0.00 |  |
| γ-terpinene | 0.00 | 0.00 | 0.00 | 0.00 | 0.02 | 0.00 | 0.00 | 0.23 | 0.00 | 0.30 | 0.00 | 0.00 | 0.00 | | 0.00 | 0.00 | 0.18 | 0.01 |  |
| α-terpinolene | 0.05 | 0.00 | 0.00 | 0.00 | 0.00 | 0.00 | 0.01 | 0.05 | 0.07 | 0.12 | 0.00 | 0.00 | 0.01 | | 0.57 | 0.00 | 0.07 | 0.00 |  |
| allocymene | 0.18 | 0.00 | 0.00 | 0.00 | 0.00 | 0.00 | 0.00 | 0.00 | 0.01 | 0.01 | 0.00 | 0.00 | 0.00 | | 0.00 | 0.00 | 0.01 | 0.00 |  |
| 1,8-cineole | 1.76 | 0.01 | 0.00 | 0.00 | 0.02 | 0.01 | 0.01 | 0.05 | 0.18 | 0.39 | 0.09 | 0.00 | 0.02 | | 0.78 | 0.00 | 0.16 | 0.01 |  |
| D-fenchyl alcohol | 0.23 | 0.00 | 0.00 | 0.00 | 0.00 | 0.00 | 0.00 | 0.00 | 0.02 | 0.02 | 0.00 | 0.00 | 0.00 | | 0.04 | 0.00 | 0.00 | 0.00 |  |
| borneol | 0.51 | 0.00 | 0.00 | 0.00 | 0.00 | 0.00 | 0.00 | 0.00 | 0.00 | 0.10 | 0.01 | 0.00 | 0.00 | | 0.09 | 0.00 | 0.00 | 0.00 |  |
| terpinen-4-ol | 3.45 | 0.00 | 0.00 | 0.00 | 0.00 | 0.00 | 0.00 | 0.00 | 0.05 | 0.16 | 0.00 | 0.00 | 0.00 | | 0.27 | 0.00 | 0.00 | 0.00 |  |
| α-terpineol | 2.81 | 0.00 | 0.00 | 0.00 | 0.00 | 0.00 | 0.00 | 0.04 | 0.10 | 0.25 | 0.00 | 0.00 | 0.00 | | 0.49 | 0.00 | 0.09 | 0.00 |  |
| geranyl acetone | 0.00 | 0.00 | 0.00 | 0.00 | 0.00 | 0.00 | 0.00 | 0.00 | 0.03 | 0.08 | 0.00 | 0.00 | 0.00 | | 0.04 | 0.00 | 0.01 | 0.00 |  |
| methyl dehydrojasmonate | 0.00 | 0.00 | 0.00 | 0.00 | 0.00 | 0.00 | 0.00 | 0.00 | 0.13 | 0.04 | 0.00 | 0.00 | 0.00 | | 0.00 | 0.00 | 0.08 | 0.00 |  |
| α-cubebene | 1.97 | 0.00 | 0.00 | 0.00 | 0.01 | 0.01 | 0.00 | 0.04 | 0.12 | 0.13 | 0.00 | 0.01 | 0.01 | | 0.00 | 0.01 | 0.00 | 0.00 |  |
| copaene | 0.00 | 0.00 | 0.00 | 0.00 | 0.00 | 0.00 | 0.01 | 0.00 | 0.00 | 0.00 | 0.01 | 0.00 | 0.00 | | 0.16 | 0.03 | 0.07 | 0.00 |  |
| caryophyllene | 0.80 | 0.00 | 0.00 | 0.00 | 0.00 | 0.00 | 0.00 | 0.00 | 0.02 | 0.03 | 0.00 | 0.00 | 0.00 | | 0.00 | 0.00 | 0.01 | 0.00 |  |
| α-humulene | 2.38 | 0.00 | 0.00 | 0.00 | 0.00 | 0.00 | 0.00 | 0.00 | 0.03 | 0.05 | 0.00 | 0.00 | 0.00 | | 0.10 | 0.00 | 0.00 | 0.00 |  |
| γ-cadinene | 2.79 | 0.00 | 0.00 | 0.00 | 0.00 | 0.00 | 0.00 | 0.01 | 0.08 | 0.04 | 0.00 | 0.00 | 0.00 | | 0.04 | 0.00 | 0.00 | 0.00 |  |
| β-selinene | 0.04 | 0.00 | 0.00 | 0.00 | 0.00 | 0.00 | 0.00 | 0.00 | 0.11 | 0.22 | 0.00 | 0.00 | 0.00 | | 0.00 | 0.00 | 0.10 | 0.00 |  |
| α-selinene | 0.23 | 0.00 | 0.00 | 0.00 | 0.00 | 0.00 | 0.00 | 0.00 | 0.08 | 0.23 | 0.01 | 0.00 | 0.01 | | 0.66 | 0.00 | 0.09 | 0.00 |  |
| α-amorphene | 1.16 | 0.00 | 0.00 | 0.00 | 0.00 | 0.00 | 0.00 | 0.00 | 0.03 | 0.00 | 0.00 | 0.00 | 0.00 | | 0.08 | 0.00 | 0.00 | 0.00 |  |
| cadinene | 4.63 | 0.00 | 0.01 | 0.00 | 0.01 | 0.01 | 0.01 | 0.09 | 0.16 | 0.15 | 0.03 | 0.01 | 0.01 | | 0.45 | 0.00 | 0.08 | 0.01 |  |
| calamenene | 0.00 | 0.00 | 0.00 | 0.00 | 0.00 | 0.00 | 0.00 | 0.00 | 0.03 | 0.05 | 0.00 | 0.00 | 0.00 | | 0.19 | 0.00 | 0.02 | 0.00 |  |
| epizonarene | 0.80 | 0.00 | 0.00 | 0.00 | 0.00 | 0.00 | 0.00 | 0.00 | 0.01 | 0.02 | 0.00 | 0.00 | 0.00 | | 0.09 | 0.00 | 0.00 | 0.00 |  |
| aromadendrene | 0.25 | 0.00 | 0.00 | 0.00 | 0.00 | 0.00 | 0.00 | 0.02 | 0.00 | 0.00 | 0.00 | 0.00 | 0.00 | | 0.02 | 0.00 | 0.00 | 0.00 |  |
| α-gurjunene | 0.06 | 0.00 | 0.00 | 0.00 | 0.00 | 0.00 | 0.00 | 0.00 | 0.01 | 0.00 | 0.00 | 0.00 | 0.00 | | 0.01 | 0.00 | 0.00 | 0.00 |  |
| α-calacorene | 0.00 | 0.00 | 0.00 | 0.00 | 0.00 | 0.00 | 0.00 | 0.01 | 0.00 | 0.02 | 0.00 | 0.00 | 0.00 | | 0.00 | 0.00 | 0.01 | 0.00 |  |
| selina-3,7(11)-diene | 0.24 | 0.00 | 0.00 | 0.00 | 0.00 | 0.00 | 0.00 | 0.00 | 0.00 | 0.02 | 0.00 | 0.00 | 0.00 | | 0.14 | 0.00 | 0.01 | 0.00 |  |
| γ-selinene | 2.29 | 0.00 | 0.00 | 0.02 | 0.00 | 0.00 | 0.00 | 0.00 | 0.01 | 0.03 | 0.00 | 0.00 | 0.00 | | 0.08 | 0.00 | 0.01 | 0.00 |  |
| aristolene | 0.00 | 0.00 | 0.00 | 0.00 | 0.00 | 0.00 | 0.00 | 0.00 | 0.00 | 0.04 | 0.00 | 0.00 | 0.00 | | 0.13 | 0.00 | 0.03 | 0.00 |  |
| β-eudesmol | 0.00 | 0.00 | 0.00 | 0.00 | 0.00 | 0.00 | 0.00 | 0.00 | 0.08 | 0.22 | 0.00 | 0.00 | 0.00 | | 0.90 | 0.00 | 0.18 | 0.00 |  |
| β-maaliene | 0.15 | 0.00 | 0.00 | 0.00 | 0.00 | 0.00 | 0.00 | 0.17 | 0.00 | 0.00 | 0.02 | 0.00 | 0.05 | | 0.01 | 0.00 | 0.12 | 0.00 |  |
| γ-eudesmol | 4.91 | 0.00 | 0.00 | 0.00 | 0.00 | 0.00 | 0.00 | 0.00 | 0.05 | 0.16 | 0.00 | 0.00 | 0.00 | | 0.96 | 0.00 | 0.00 | 0.00 |  |
| α-eudesmol | 8.80 | 0.00 | 0.00 | 0.00 | 0.00 | 0.00 | 0.00 | 0.00 | 0.00 | 0.31 | 0.00 | 0.00 | 0.00 | | 0.00 | 0.00 | 0.24 | 0.00 |  |
| cadalene | 0.51 | 0.00 | 0.00 | 0.00 | 0.00 | 0.00 | 0.00 | 0.00 | 0.06 | 0.10 | 0.00 | 0.00 | 0.00 | | 0.13 | 0.00 | 0.10 | 0.00 |  |
| p-xylene | 0.00 | 0.00 | 0.00 | 0.00 | 0.00 | 0.00 | 0.00 | 0.00 | 0.00 | 0.00 | 0.00 | 0.00 | 0.00 | | 0.10 | 0.00 | 0.00 | 0.00 |  |
| benzaldehyde | 3.88 | 0.00 | 0.00 | 0.00 | 0.00 | 0.00 | 0.00 | 0.00 | 0.45 | 0.67 | 0.00 | 0.00 | 0.00 | | 0.61 | 0.00 | 0.00 | 0.00 |  |
| 1,4-dimethoxybenzene | 0.60 | 0.00 | 0.00 | 0.00 | 0.00 | 0.00 | 0.00 | 0.00 | 0.08 | 0.09 | 0.00 | 0.00 | 0.00 | | 0.07 | 0.00 | 0.07 | 0.00 |  |
| 2-methylnaphthalene | 0.00 | 0.00 | 0.00 | 0.00 | 0.00 | 0.00 | 0.00 | 0.00 | 0.00 | 0.02 | 0.00 | 0.00 | 0.00 | | 0.01 | 0.00 | 0.01 | 0.00 |  |
| 6h-furo [2',3':4,5]oxazolo [3,2-a]pyrimidin-6-one,  3-(acetyloxy)-2,3,3a,9a-tetrahydro-2-  [[(trimethylsilyl)oxy]methyl]- | 0.14 | 0.00 | 0.00 | 0.00 | 0.00 | 0.00 | 0.00 | 0.01 | 0.00 | 0.00 | 0.00 | 0.00 | 0.00 | | 0.00 | 0.00 | 0.00 | 0.00 |  |
| cyclohexylbenzene | 0.04 | 0.00 | 0.00 | 0.00 | 0.00 | 0.00 | 0.00 | 0.00 | 0.00 | 0.09 | 0.00 | 0.00 | 0.00 | | 0.03 | 0.00 | 0.00 | 0.00 |  |
| benzoicacid,butylester | 0.00 | 0.00 | 0.00 | 0.00 | 0.00 | 0.00 | 0.00 | 0.00 | 0.17 | 0.35 | 0.00 | 0.00 | 0.00 | | 0.84 | 0.00 | 0.23 | 0.00 |  |
| 2,6-dimethylnaphthalene | 0.00 | 0.00 | 0.00 | 0.00 | 0.00 | 0.00 | 0.00 | 0.00 | 0.00 | 0.01 | 0.00 | 0.00 | 0.00 | | 0.00 | 0.00 | 0.00 | 0.00 |  |
| butylated hydroxytoluene | 0.00 | 0.00 | 0.00 | 0.00 | 0.00 | 0.00 | 0.00 | 0.00 | 0.00 | 0.02 | 0.00 | 0.00 | 0.00 | | 0.05 | 0.00 | 0.00 | 0.00 |  |
| 1h-indene | 0.12 | 0.00 | 0.00 | 0.00 | 0.00 | 0.00 | 0.00 | 0.20 | 0.00 | 0.00 | 0.04 | 0.00 | 0.00 | | 0.00 | 0.00 | 0.00 | 0.00 |  |
| 2,6-diisopropylnaphthalene | 0.00 | 0.00 | 0.00 | 0.00 | 0.00 | 0.00 | 0.00 | 0.00 | 0.06 | 0.06 | 0.00 | 0.00 | 0.00 | | 0.02 | 0.00 | 0.06 | 0.00 |  |
