# Supplementary material for: Influence of increased nutrient availability on biogenic volatile organic compound (BVOC) emissions and leaf anatomy of subarctic dwarf shrubs under climate warming and increased cloudiness
Source: Ann Bot. 2022 Jan 13;129(4):443–55. doi: 10.1093/aob/mcac004 (PMC8944702; doi:10.1093/aob/mcac004)
Supplement: mcac004_suppl_Supplementary_Table_S6 [file mcac004_suppl_supplementary_table_s6.docx]

Table S6. Emissions (µg g^-1^ h^-1^) of individual compounds from *B. nana* under long-term control (C), shading (S), and warming (W) treatments.

|  | Control (C) | | | | | | Shading (S) | | | | | Warming (W) | | | | | |
| --- | --- | --- | --- | --- | --- | --- | --- | --- | --- | --- | --- | --- | --- | --- | --- | --- | --- |
|  | C | C | C | C | C | C | S | S | S | S | W | | W | W | W | W |  |
| isoprene | 0.80 | 0.03 | 0.00 | 0.04 | 0.00 | 0.12 | 1.06 | 0.12 | 0.00 | 0.02 | 1.54 | | 0.49 | 0.01 | 0.03 | 0.03 |  |
| cis-3-hexen-1-ol | 14.46 | 0.00 | 0.00 | 0.00 | 0.30 | 0.00 | 38.28 | 0.00 | 0.86 | 0.28 | 9.06 | | 0.72 | 0.00 | 0.00 | 0.00 |  |
| cis-2-hexen-1-ol | 2.99 | 0.00 | 0.00 | 0.00 | 0.00 | 0.00 | 14.90 | 0.00 | 0.00 | 0.00 | 0.00 | | 0.00 | 0.00 | 0.00 | 0.00 |  |
| cis-3-hexenyl acetate | 24.34 | 0.48 | 0.00 | 0.00 | 0.00 | 0.37 | 22.25 | 0.00 | 0.00 | 0.00 | 36.74 | | 1.44 | 0.00 | 0.33 | 0.00 |  |
| α-pinene | 0.00 | 0.00 | 0.00 | 0.00 | 0.00 | 0.00 | 0.00 | 0.01 | 0.00 | 0.02 | 0.03 | | 0.00 | 0.00 | 0.05 | 0.00 |  |
| α-phellandrene | 0.00 | 0.01 | 0.00 | 0.00 | 0.45 | 0.00 | 0.00 | 0.00 | 0.00 | 0.00 | 0.00 | | 0.00 | 0.00 | 0.00 | 0.00 |  |
| limonene | 0.15 | 0.00 | 0.00 | 0.00 | 0.00 | 0.00 | 0.05 | 0.00 | 0.00 | 0.00 | 0.31 | | 0.00 | 0.00 | 0.01 | 0.00 |  |
| β-ocimene | 0.00 | 0.00 | 0.00 | 0.00 | 0.00 | 0.00 | 0.02 | 0.00 | 0.00 | 0.00 | 0.08 | | 0.00 | 0.00 | 0.00 | 0.00 |  |
| cymenene | 0.04 | 0.00 | 0.00 | 0.00 | 0.00 | 0.00 | 0.04 | 0.00 | 0.00 | 0.00 | 0.07 | | 0.00 | 0.00 | 0.00 | 0.00 |  |
| 1,8-cineole | 0.00 | 0.00 | 0.00 | 0.00 | 0.00 | 0.00 | 0.24 | 0.00 | 0.00 | 0.01 | 0.00 | | 0.00 | 0.00 | 0.00 | 0.00 |  |
| terpineol | 0.00 | 0.00 | 0.00 | 0.00 | 0.00 | 0.00 | 0.00 | 0.00 | 0.00 | 0.00 | 0.17 | | 0.00 | 0.00 | 0.00 | 0.00 |  |
| bornylacetate | 0.07 | 0.00 | 0.00 | 0.00 | 0.00 | 0.00 | 0.05 | 0.00 | 0.00 | 0.00 | 0.07 | | 0.00 | 0.00 | 0.00 | 0.00 |  |
| geranylacetone | 0.18 | 0.00 | 0.00 | 0.00 | 0.00 | 0.00 | 0.00 | 0.00 | 0.00 | 0.00 | 0.18 | | 0.00 | 0.00 | 0.00 | 0.00 |  |
| ylangene | 0.09 | 0.00 | 0.00 | 0.00 | 0.00 | 0.00 | 0.11 | 0.00 | 0.00 | 0.00 | 0.11 | | 0.00 | 0.00 | 0.00 | 0.00 |  |
| copaene | 0.05 | 0.00 | 0.00 | 0.00 | 0.00 | 0.00 | 0.25 | 0.00 | 0.00 | 0.04 | 0.40 | | 0.00 | 0.00 | 0.00 | 0.00 |  |
| β-bourbonene | 0.00 | 0.00 | 0.00 | 0.00 | 0.00 | 0.00 | 0.00 | 0.00 | 0.00 | 0.04 | 0.00 | | 0.00 | 0.00 | 0.00 | 0.00 |  |
| caryophyllene | 0.03 | 0.00 | 0.00 | 0.00 | 0.02 | 0.00 | 0.08 | 0.00 | 0.00 | 0.01 | 0.08 | | 0.05 | 0.00 | 0.00 | 0.00 |  |
| α-humulene | 0.02 | 0.00 | 0.00 | 0.00 | 0.00 | 0.00 | 0.03 | 0.00 | 0.00 | 0.00 | 0.03 | | 0.00 | 0.00 | 0.00 | 0.00 |  |
| alloaromadendrene | 0.00 | 0.00 | 0.00 | 0.00 | 0.00 | 0.00 | 0.45 | 0.00 | 0.00 | 0.06 | 0.90 | | 0.00 | 0.00 | 0.00 | 0.00 |  |
| α-selinene | 0.32 | 0.00 | 0.00 | 0.00 | 0.06 | 0.00 | 0.12 | 0.00 | 0.00 | 0.00 | 0.84 | | 0.00 | 0.00 | 0.00 | 0.00 |  |
| benzaldehyde | 1.42 | 0.00 | 0.00 | 0.00 | 0.00 | 0.00 | 0.04 | 0.00 | 0.00 | 0.00 | 0.91 | | 0.00 | 0.00 | 0.00 | 0.00 |  |
| acetophenone | 0.32 | 0.00 | 0.00 | 0.00 | 0.00 | 0.00 | 0.43 | 0.00 | 0.00 | 0.00 | 0.34 | | 0.00 | 0.00 | 0.00 | 0.00 |  |
