# Supplementary material for: Influence of increased nutrient availability on biogenic volatile organic compound (BVOC) emissions and leaf anatomy of subarctic dwarf shrubs under climate warming and increased cloudiness
Source: Ann Bot. 2022 Jan 13;129(4):443–55. doi: 10.1093/aob/mcac004 (PMC8944702; doi:10.1093/aob/mcac004)
Supplement: mcac004_suppl_Supplementary_Table_S7 [file mcac004_suppl_supplementary_table_s7.docx]

Table S7. Emissions (µg g^-1^ h^-1^) of individual compounds from *B. nana* under long-term fertilization (F), fertilization + shading (FS), and fertilization + warming (FW) treatments.

|  | Fertilization (F) | | | | | Fertilization + Shading (FS) | | | | | | | Fertilization + Warming (FW) | | | | |
| --- | --- | --- | --- | --- | --- | --- | --- | --- | --- | --- | --- | --- | --- | --- | --- | --- | --- |
|  | F | F | F | F | F | FS | FS | FS | FS | FS | FS | FS | FW | FW | FW | FW | FW |
| isoprene | 0.00 | 0.00 | 0.00 | 0.24 | 0.00 | 0.00 | 0.00 | 10.07 | 0.07 | 0.00 | 0.00 | 0.00 | 0.05 | 0.00 | 0.03 | 0.00 | 0.00 |
| cis-3-hexen-1-ol | 0.00 | 14.34 | 0.94 | 0.88 | 0.00 | 0.00 | 1.86 | 3.04 | 0.00 | 9.57 | 0.00 | 2.01 | 0.44 | 0.36 | 0.00 | 5.67 | 0.48 |
| cis-2-hexen-1-ol | 0.00 | 5.53 | 0.22 | 0.00 | 0.00 | 0.00 | 0.35 | 0.00 | 0.00 | 0.00 | 0.00 | 0.00 | 0.02 | 0.00 | 0.00 | 0.90 | 0.00 |
| cis-3-hexenyl acetate | 0.00 | 31.65 | 0.00 | 0.00 | 0.00 | 0.00 | 0.00 | 0.00 | 0.00 | 0.00 | 0.30 | 0.00 | 0.00 | 0.00 | 0.39 | 0.00 | 0.00 |
| cis-2-hexenyl acetate | 0.00 | 0.00 | 0.05 | 0.00 | 0.00 | 0.00 | 0.40 | 0.29 | 0.00 | 0.00 | 0.00 | 0.00 | 0.00 | 0.16 | 0.00 | 0.00 | 0.00 |
| α-pinene | 0.07 | 0.00 | 0.00 | 0.00 | 0.00 | 0.00 | 0.00 | 0.01 | 0.06 | 0.00 | 0.00 | 0.00 | 0.00 | 0.00 | 0.00 | 0.00 | 0.00 |
| α-phellandrene | 0.00 | 0.00 | 0.14 | 1.64 | 0.00 | 0.00 | 0.71 | 1.09 | 0.00 | 1.38 | 0.00 | 0.43 | 0.46 | 0.87 | 0.00 | 0.92 | 0.32 |
| limonene | 0.01 | 0.16 | 0.00 | 0.00 | 0.00 | 0.00 | 0.00 | 0.01 | 0.01 | 0.00 | 0.00 | 0.00 | 0.00 | 0.00 | 0.00 | 0.00 | 0.00 |
| β-ocimene | 0.00 | 0.07 | 0.00 | 0.00 | 0.00 | 0.00 | 0.01 | 0.01 | 0.00 | 0.00 | 0.00 | 0.00 | 0.00 | 0.00 | 0.00 | 0.00 | 0.00 |
| cymenene | 0.00 | 0.04 | 0.00 | 0.00 | 0.00 | 0.00 | 0.00 | 0.00 | 0.00 | 0.00 | 0.00 | 0.00 | 0.00 | 0.00 | 0.00 | 0.00 | 0.00 |
| 1,8-cineole | 0.01 | 0.00 | 0.00 | 0.00 | 0.00 | 0.00 | 0.00 | 0.01 | 0.03 | 0.00 | 0.00 | 0.00 | 0.00 | 0.00 | 0.00 | 0.00 | 0.00 |
| terpineol | 0.00 | 0.00 | 0.02 | 0.00 | 0.00 | 0.00 | 0.04 | 0.03 | 0.00 | 0.07 | 0.00 | 0.04 | 0.04 | 0.01 | 0.00 | 0.09 | 0.01 |
| bornylacetate | 0.00 | 0.07 | 0.00 | 0.00 | 0.00 | 0.00 | 0.00 | 0.00 | 0.00 | 0.00 | 0.00 | 0.00 | 0.00 | 0.00 | 0.00 | 0.00 | 0.00 |
| geranylacetone | 0.00 | 0.15 | 0.07 | 0.00 | 0.00 | 0.00 | 0.00 | 0.05 | 0.00 | 0.00 | 0.00 | 0.00 | 0.00 | 0.00 | 0.00 | 0.00 | 0.00 |
| ylangene | 0.00 | 0.10 | 0.03 | 0.00 | 0.00 | 0.02 | 0.00 | 0.00 | 0.00 | 0.00 | 0.00 | 0.00 | 0.11 | 0.00 | 0.00 | 0.00 | 0.00 |
| copaene | 0.00 | 0.37 | 0.00 | 0.00 | 0.00 | 0.00 | 0.00 | 0.48 | 0.00 | 0.00 | 0.00 | 0.00 | 0.00 | 0.00 | 0.00 | 0.00 | 0.01 |
| β-bourbonene | 0.00 | 0.10 | 0.01 | 0.00 | 0.00 | 0.01 | 0.00 | 0.00 | 0.00 | 0.00 | 0.00 | 0.00 | 0.00 | 0.00 | 0.00 | 0.00 | 0.02 |
| caryophyllene | 0.00 | 0.23 | 0.01 | 0.06 | 0.00 | 0.02 | 0.00 | 0.15 | 0.00 | 0.36 | 0.00 | 0.00 | 0.03 | 0.00 | 0.00 | 0.00 | 0.00 |
| α-humulene | 0.00 | 0.03 | 0.00 | 0.00 | 0.00 | 0.00 | 0.00 | 0.07 | 0.00 | 0.10 | 0.01 | 0.00 | 0.01 | 0.00 | 0.00 | 0.00 | 0.00 |
| alloaromadendrene | 0.00 | 0.62 | 0.05 | 0.00 | 0.00 | 0.00 | 0.00 | 0.88 | 0.00 | 0.00 | 0.00 | 0.00 | 0.21 | 0.00 | 0.00 | 0.00 | 0.03 |
| α-selinene | 0.00 | 0.32 | 0.00 | 0.00 | 0.00 | 0.00 | 0.00 | 0.00 | 0.04 | 0.00 | 0.05 | 0.00 | 0.00 | 0.00 | 0.00 | 0.00 | 0.00 |
| benzaldehyde | 0.00 | 1.34 | 0.00 | 0.00 | 0.00 | 0.00 | 0.00 | 0.00 | 0.00 | 0.00 | 0.00 | 0.00 | 0.00 | 0.00 | 0.00 | 0.00 | 0.00 |
| acetophenone | 0.00 | 0.87 | 0.00 | 0.00 | 0.00 | 0.00 | 0.00 | 0.00 | 0.00 | 0.00 | 0.00 | 0.00 | 0.00 | 0.00 | 0.00 | 0.00 | 0.00 |
|  |  |  |  |  |  |  |  |  |  |  |  |  |  |  |  |  |  |
